# The prevalence of dyslipidemia and its correlation with anti-retroviral therapy among people living with HIV in China: a systematic review and meta-analysis

**DOI:** 10.3389/fcvm.2025.1498165

**Published:** 2025-06-13

**Authors:** Zhai Wenjing, Ouyang Fei, Zhan Shenao, Huang Yu, Fu Gengfeng, Yang Haitao

**Affiliations:** ^1^ Key Laboratory of Environmental Medicine Engineering of Ministry of Education, Department of Epidemiology and Health Statistics, School of Public Health, Southeast University, Nanjing, China; ^2^ Department of Epidemiology and Biostatistics, School of Public Health, Nanjing Medical University, Nanjing, Jiangsu, China; ^3^ Jiangsu Provincial Center for Disease Control and Prevention, Nanjing, China

**Keywords:** HIV, anti-retroviral therapy, China, meta-analysis, dyslipidemia

## Abstract

**Background:**

Dyslipidemia, a risk factor of cardiovascular diseases, was a long-term adverse event of anti-retroviral drugs. Efavirenz (EFV) and lopinavir/ritonavir (LPV/r) were recommended and the widely used antiretroviral drugs while the proportion of taking integrase strand transfer inhibitors (INSTI)-based regimens are increasing recently in China. Regarding to the large population of people living with HIV (PLWH) in China and the regional fluctuations in prevalence of dyslipidemia, this meta-analysis aims to evaluate the prevalence of dyslipidemia and its correlation with anti-retroviral therapy (ART) among PLWH in China, especially the impact of LPV/r, EFV and INSTI-based regimens.

**Methods:**

We searched English and Chinese databases using MeSH terms to identify all relevant articles. The study participants were divided into ART-naïve and ART-experienced PLWH. The prevalence of dyslipidemia and mean difference of serum lipids were estimated using random-effects models. Subgroup analysis and univariate meta-regression were conducted to evaluate factors associated with prevalence of dyslipidemia among ART-experienced PLWH.

**Results:**

In this meta-analysis, we found dyslipidemia prevalence of 49.8% and 55.1% among ART-naïve and experienced PLWH in China. Elevated triglycerides(TG) and reduced high-density lipoprotein cholesterol (HDL-C) were the most prevalent dyslipidemia, irrespective of ART experience. Dyslipidemia was more common in PLWH residing in South China, with baseline CD4 cell count over 500 cells/μl or with a BMI ≥ 24 kg/m^2^. Notably, Traditional Chinese medicine adjuvant therapy was associated with higher prevalence of dyslipidemia. Moreover, INSTI-based regimens were significantly linked to higher prevalence of low HDL-C compared to other regimens.

**Conclusions:**

The routine assessment of lipid profiles should be advised among PLWH before and after the initiation of ART in China, especially in patients on INSTI-based regiments. Moreover, early interventions, including physical activity, dietary adjustments, and optimization of ART regimens, should be considered when the dyslipidemia is diagnosed in PLWH.

## 1 Introduction

Anti-retroviral therapy (ART) has led to a significant reduction in mortality among individuals living with HIV and a marked increase in life expectancy over the past few decades (1, 2). This has led to a shift in morbidity and mortality patterns among people living with HIV (PLWH), from predominantly AIDS-related illnesses to non-communicable comorbidities (3). Long-term cohort revealed that cardiovascular disease (CVD) and cancer are the pre-dominant cause of death in non-AIDS-related deaths worldwide, which has also been the leading cause of death among Chinese PLWH (2, 4). Dyslipidemia, a notable long-term adverse effect of ART, is a significant contributor to CVD-related mortality and is widely recognized as one of the most prevalent risk factors for CVD (5, 6).

Dyslipidemia typically refers to abnormal serum levels of total cholesterol (TC) or TG, but broadly includes elevated TC, TG, low-density lipoprotein cholesterol (LDL-C) or a low serum concentration of HDL-C or a combination of these features (7).

Diet rich in saturated fatty acids and lack of physical activity are important risk factors for dyslipidemia (8). Besides, HIV infection, antiretroviral treatment and chronic inflammation can also impact lipid profiles. HIV infection has been linked to increases in atherogenic lipids, such as LDL-C and triglycerides, alongside decreases in both HDL levels and function (9). Macrophages in the arterial wall utilize ATP-binding cassette (ABC) transporters, such as ABCA1, to transfer atherogenic lipids to HDL or apolipoprotein A1, facilitating reverse cholesterol transport and cholesterol efflux. In an *in vitro* model, monocytes from HIV-positive donors exhibited lower ABCA1 gene expression. Additionally, monocytes exposed to pooled serum from ART-treated HIV-positive individuals were more likely to become foam cells compared to those exposed to serum from HIV-negative donors, indicating that both HIV infection and ART may impair cholesterol efflux (10).

China is a country with low prevalence of HIV, but there were still around 1.40 million PLWH and AIDS by October of 2024 (11). Free HIV antiretroviral treatment and a “test-and-treat” policy were implemented in China since 2003, enabling over 90% of HIV-infected individuals to receive antiretroviral therapy (12). The antiretroviral regimen commonly used in China combines nucleoside reverse transcriptase inhibitors (NRTIs) with a third drug, with LPV/r and EFV being the main choices since 2011 (13). LPV/r-based regimens are widely used as second-line antiretroviral therapy in China and EFV-based regimens are recommended as first-line antiretroviral therapy for newly discovered HIV infected individuals (14). However, the inclusion of INSTI in China's national reimbursement drug list in 2021, with price reductions and favorable antiretroviral efficacy, has attracted more newly-discovered HIV-infected patients to choose INSTI-based regimen as their treatment (15, 16).

**Figure 1:**
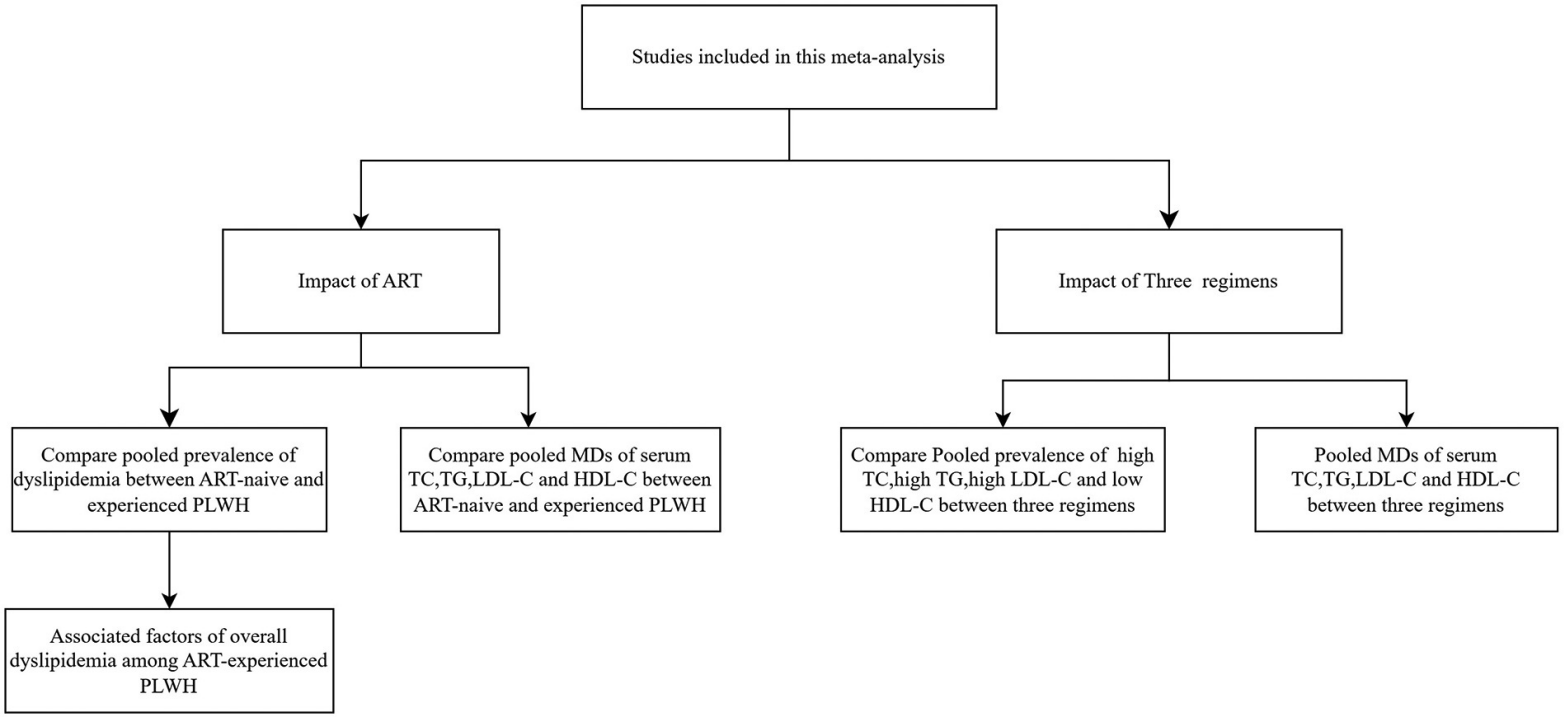
Meta-analysis study flowchart on dyslipidemia among HIV-infected individuals in China.

Therefore, it is essential to evaluate the impact of LPV/r-based, EFV-based and INSTI-based regimens on serum levels of lipids. Besides, the prevalence of dyslipidemia among Chinese PLWH were reported to fluctuate widely from 20% to 80% across regions (17–19). Moreover, studies related to dyslipidemia in Chinese PLWH were characterized by small sample sizes and short observation periods. Thus, this study aims to assess the impact of antiretroviral therapy and ART regimens (LPV/r-based or EFV-based or INSTI-based) on dyslipidemia. The primary focus is to compare the prevalence and levels of four types of lipid abnormalities before and after ART, and to conduct subgroup analyses to identify factors associated with dyslipidemia. The secondary objective is to compare the prevalence and levels of lipid abnormalities among three different ART regimens (Figure 1).

## 2 Methods

### 2.1 Search strategy

The research was conducted in accordance with Preferred Reporting Items for Systematic Reviews and Meta-analyses (PRISMA) (Supplementary Table S1) (20). We searched for articles on population-based studies about prevalence of dyslipidemia in Chinese PLWH using English databases PubMed, Embase, Web of Science, and Chinese databases CNKI (Chinese National Knowledge Infrastructure), CBM (Chinese Biomedical Literature Database), VIP (China Science and Technology Journal Database) and Wanfang electronic databases.

The search strategy consisted of terms such as ART, HIV, dyslipidemia, hyperlipidemias, hypercholesterolemia, hypertriglyceridemia, total cholesterol, triglycerides, LDL-C and HDL-C. The search terms for PubMed and CNKI were listed in Supplementary Tables S2, S3. This search was restricted to articles conducted in China and published from the earliest year provided by databases to 1st March 2025.

### 2.2 Study selection and inclusion and exclusion criteria

Two reviewers independently completed the identification, screening, and inclusion of studies using Endnote 20 and Zotero 7. Any disputes in these processes were discussed and resolved with a third reviewer.

Inclusion criteria were:(1) Study population included ART-naïve PLWH and PLWH experiencing ART over 6 months in China; (2) Reporting prevalence of dyslipidemia in ART-experienced or both ART-experienced and naïve groups; (3) Reporting mean serum levels and standard deviations (SD) of TC, TG, LDL-C and HDL-C in both ART-experienced and naïve groups; (4) If there were several articles based on the same participants, the one with more detailed data would be selected. Exclusion criteria were: (1) Meta-analysis, reviews, guidelines, case-reports, animal experiments, conference and meeting abstracts; (2) Prevalence of dyslipidemia or mean levels and SDs of serum lipids not clearly reported or duplicated; (3) Control group not based on ART-naïve PLWH; (4) Study population including pregnant or lactating women and children aged under 15; (5) Studies reporting mean levels of serum lipids including patients with heart failure, diabetes, hypertension or tumor; (6) Sample size less than 100 in studies reporting prevalence of dyslipidemia; (7) Diagnostic criteria of dyslipidemia not based on National Cholesterol Education Program Adult Treatment Panel III (NCEP-ATP III) or Guidelines on Prevention and Treatment of Blood Lipid Abnormality in Chinese Adults (2016); (8) Low quality and high risk of bias studies.

### 2.3 Data extraction and assessment of risk of bias

Two reviewers independently completed data extraction and the risk of bias evaluation. Any difference in these processes was discussed and determined with a third reviewer. The extracted information included: (1) Article citations (name of first author and year of publication); (2) Study characteristics (region, study design, time of recruitment, study population and sample size); (3) Basic characteristics of patients (mean, median or range of age, gender, proportion of overweight or diabetes or hyper-tension at baseline, history of smoking, body mass index (BMI), years since HIV infected, duration of ART, ART regimens, CD4 + T-cell counts at baseline, taking traditional Chinese Medicine (TCM) therapy to assist antiretroviral therapy; (4) Outcomes: prevalence of dyslipidemia (numbers of total study population, number of cases of dyslipidemia and number of cases of high TC, high TG, high LDL-C or low HDL-C in both groups) and means and SDs of serum lipids levels (TC, TG, LDL-C or HDL-C) in both groups. When the information was not directly available, it was calculated if possible.

In this research, the potential bias of the prevalent studies was assessed by the Joanna Briggs Institute (JBI) scales (21). The studies were classified as low-quality and high-risk of bias if the total score was ≤4 (22). The Newcastle-Ottawa Scale for cohorts and the revised JBI critical appraisal tool for the assessment of risk of bias for randomized controlled trials were used for cohorts and randomized controlled trials reporting serum levels of lipids (Supplementary Table S4–S6) (23, 24).

### 2.4 Statistical analysis

Analyses were performed using R software (version 4.2.2) with the “meta” and “metafor” packages. Two-tailed *p*-values less than 0.05 were considered statistically significant, unless otherwise specified. Effect estimates were reported with 95% confidence intervals and exact *p*-values. Heterogeneity across studies was evaluated using Cochran's Q statistic and I^2^ statistic, where a *p*-value ≤ 0.1 or I^2^ ≥ 50% indicated significant heterogeneity (25).

The random effects model was chosen to estimate the prevalence of dyslipidemia and mean differences of serum lipids in Chinese PLWH due to high heterogeneity between included studies and forest plots were used for visualization (26). Chi-square tests were used to compare the pooled prevalence of dyslipidemia (over-all, high TC, high TG, high LDL-C, and low HDL-C) between ART-naïve and experienced PLWH (27, 28).

Funnel plots and Egger's test were used to assess publication bias (29). When publication bias was apparent (*p* < 0.05), we ascertained its potential impact on the pooled estimates using the “trim and fill” analysis of Duval and Tweedie (30). Leave-one-study-out sensitivity analysis was performed to determine the stability of the results (31).

### 2.5 Definitions of subgroup design

To explore factors associated with the overall prevalence of dyslipidemia in ART-experienced PLWH, we conducted subgroup analyses based on region (North or South), study design (cross-sectional or cohort), age (<50 or ≥50 years), gender (Male or Female), body mass index (BMI: < 18.5, 18.5–23.9, or ≥24), years since HIV infection (≤5 or >5 years), duration of ART (≤5 or >5 years), baseline CD4^+^ T cell count (≤350, 350–500, or >500 cells/μl), fasting blood glucose (FPG: < 6.1 or ≥6.1 mmol/L), use of Traditional Chinese Medicine therapy (Yes or No), and ART regimens (LPV/r-based or EFV-based or INSTI-based). Subgroup analysis variables were included in this meta-analysis based on the availability of subgroup data in the enrolled literature, provided that at least two studies reported the relevant subgroup. Meanwhile, univariate meta-regression analysis was performed for all variables and was conducted to analyze the effects of EFV-based, LPV/r-based and INSTI-based regimens on prevalence of dyslipidemia and MDs of serum lipids when there were more than 2 studies reported related information (32).

## 3 Results

### 3.1 Literature screening and characteristics of included studies

**Figure 2:**
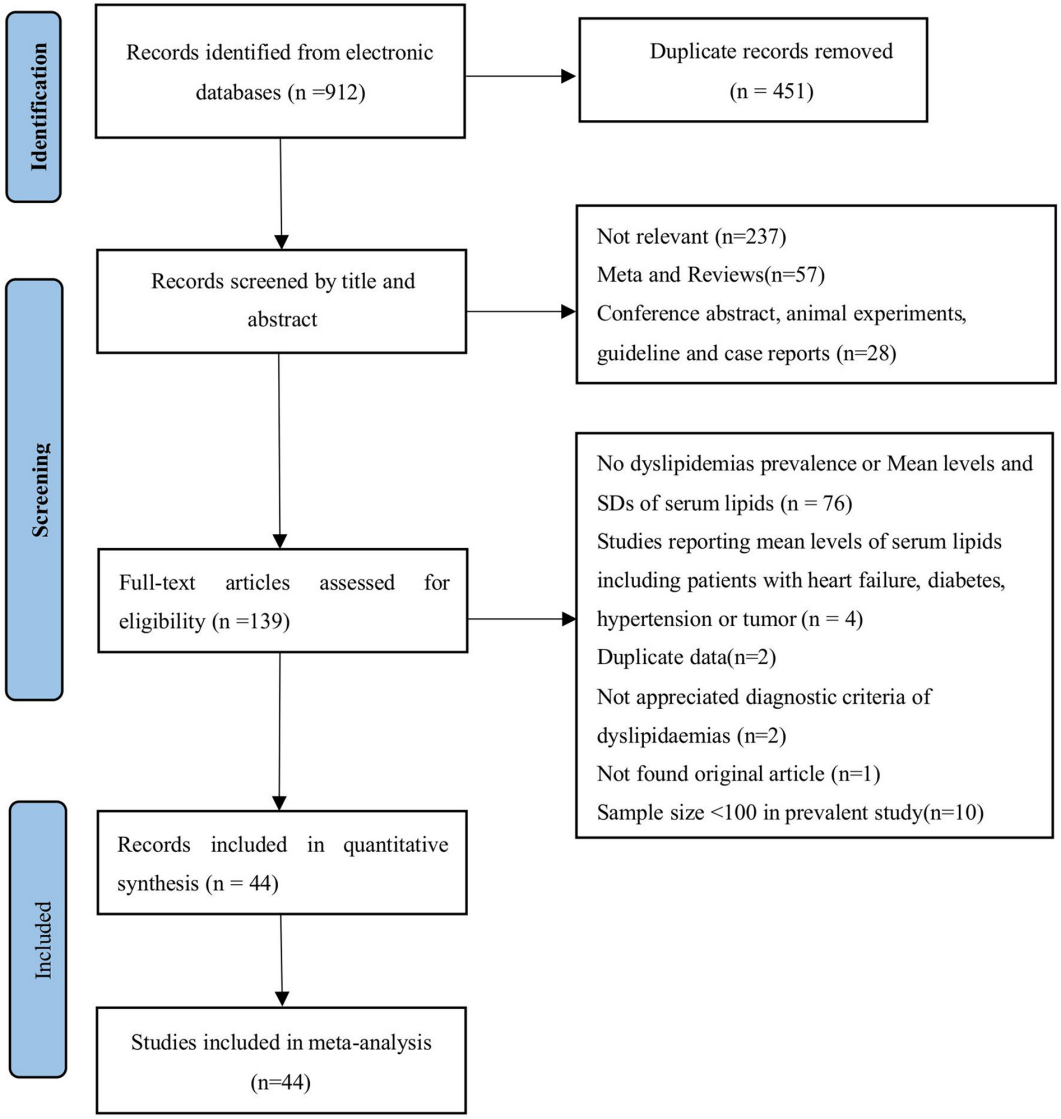
PRISMA flow diagram showing study selection for meta-analysis of dyslipidaemias and the correlates of anti-retroviral therapy among people living with HIV in China.

The initial online search yielded 461 potential studies after removing duplicated records (Figure 2). Upon screening titles and abstracts, 139 studies underwent assessment for eligibility through full-text articles. Of these, 94 articles were excluded for non-compliance with requirements. The scores of the risk of bias evaluation in the included studies were 5 to 9 points (Supplementary Tables S4–S7).

The current meta-analysis finally comprised 45 studies (32 articles only reporting prevalence, 9 only reporting MDs of lipids and 4 reporting both). These studies included 52,463 HIV-infected patients. The research region covered 14 provinces, that were 5 northern provinces (Gansu, Xinjiang, Henan, Hubei and Beijing) and 9 southern provinces (Zhejiang, Jiangsu, Fujian, Guangdong, Guangxi, Sichuan, Yunnan, Shanghai and Chongqing) in China (More information of included studies in Supplementary Table S8).

### 3.2 Overall prevalence of dyslipidemia among PLWH

**Figure 3:**
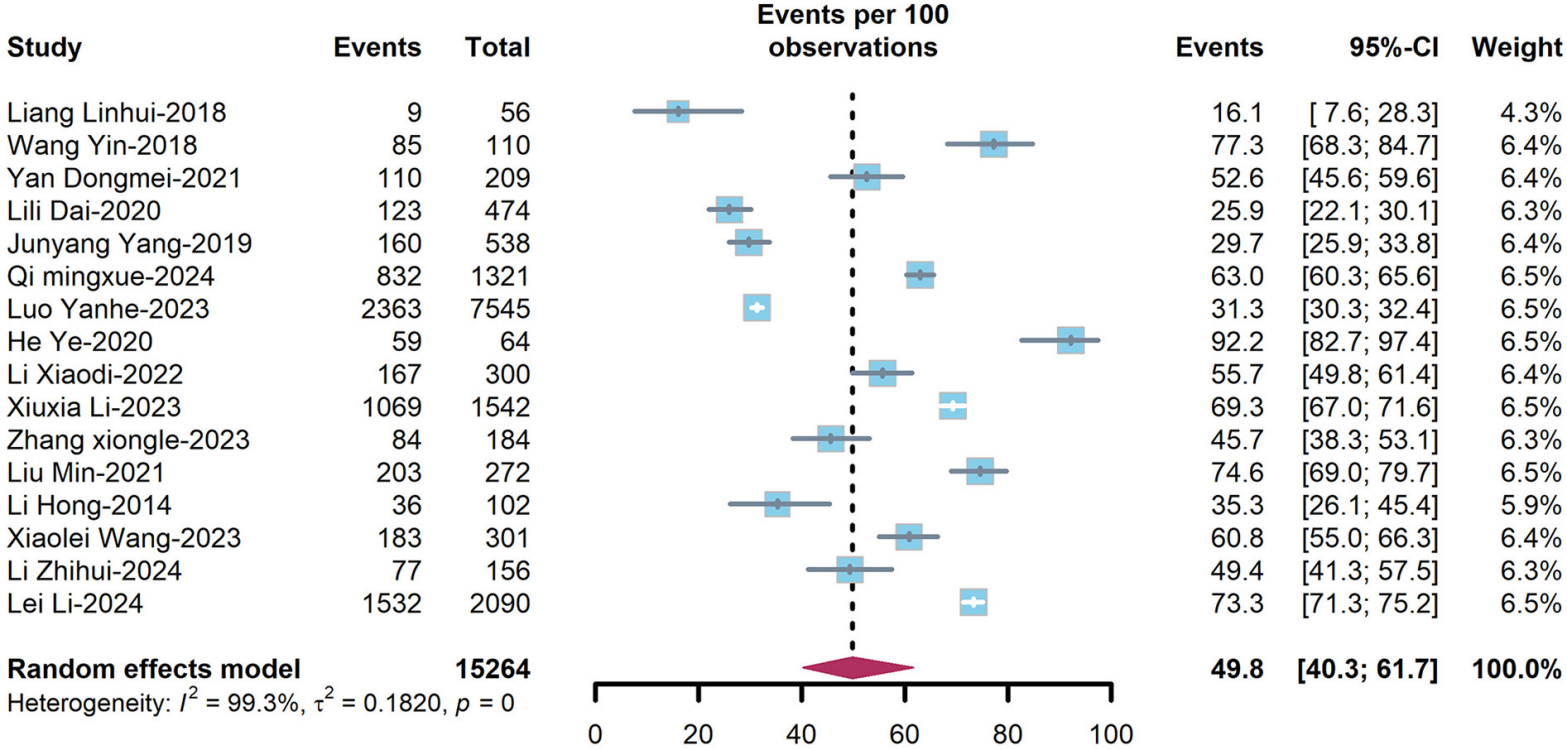
Forest plot of prevalence of dyslipidaemias among ART-naïve PLWH.

**Figure 4:**
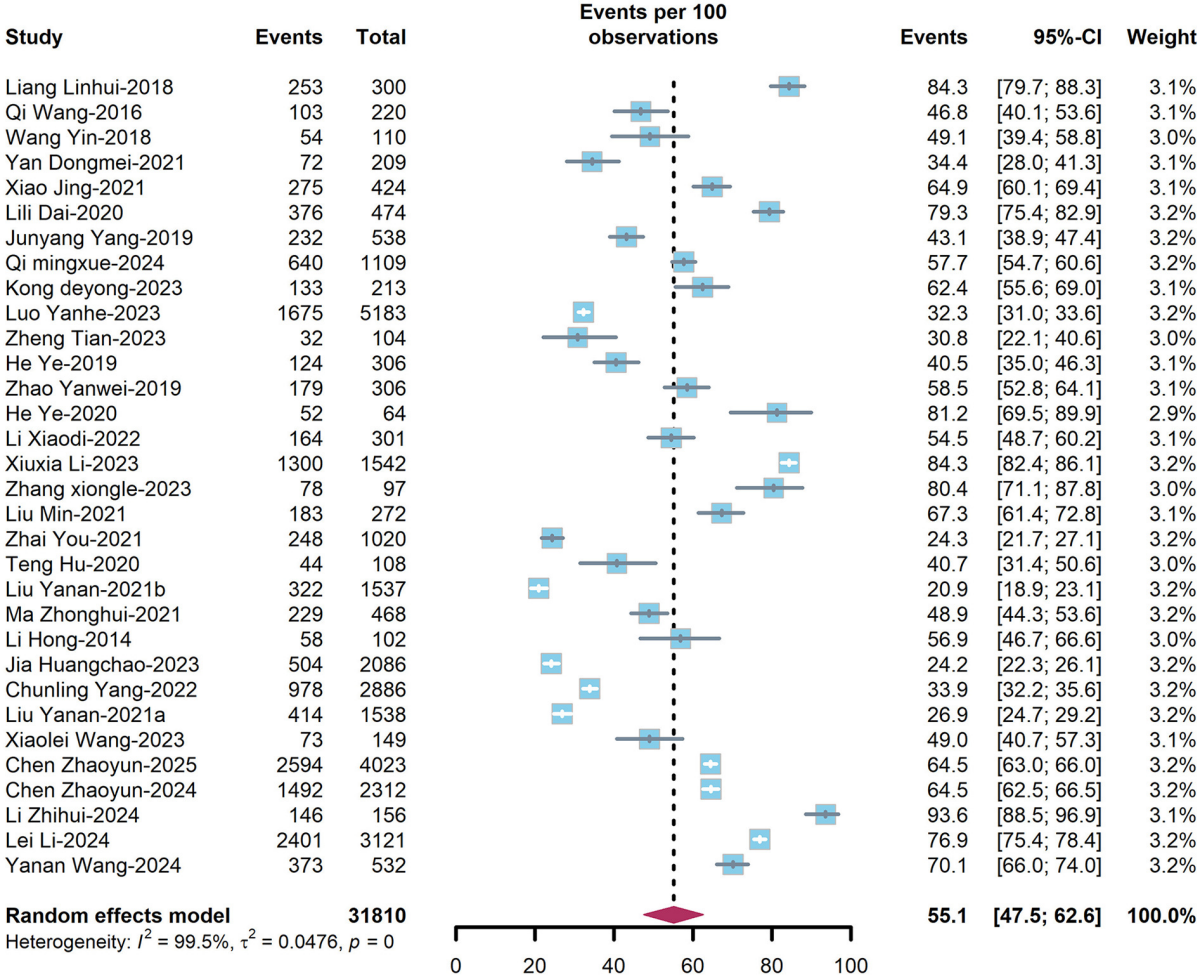
Forest plot of prevalence of dyslipidaemias among ART-experienced PLWH.

The overall prevalence of dyslipidemia among PLWH in China was estimated at 49.8% (95%CI: 40.3–61.7%) (Figure 3) in treatment-naïve individuals and 55.1% (95%CI: 47.5–62.6%) (Figure 4) in ART-experienced patients. The chi-square analysis revealed a statistically significant difference in overall prevalence of dyslipidemia between two groups (*χ*^2^ = 2137.9, *p* < 0.001) (Table 1).

**Table 1:** Prevalence of dyslipidemia before and after initiating ART.

Lipid abnormality type	Group	No. of Study	Sample size	Pooled prevalence (%) (95%CI)	Q	Heterogeneity		Chisq-Test	
						*I* ^2^	*p*	*χ* ^2^	*p*
Dyslipidemia	Naïve	16	15,264	49.8 (40.25–61.7)	2,243.8	99.3%	<0.01	2,137.9	<0.01
	ART	32	31,810	55.1 (47.5–62.6)	6,168.2	99.3%	<0.01		
High TC	Naïve	13	6,881	11.1 (8.3–15.0)	144.3	91.7%	<0.01	29,529.0	<0.01
	ART	25	28,188	23.5 (15.8–32.2)	3,593.5	99.3%	<0.01		
High TG	Naïve	12	6,609	22.6 (16.3–29.4)	185.1	94.1%	<0.01	26,862.9	<0.01
	ART	25	28,367	40.6 (31.6–49.9)	2,110.8	98.9%	<0.01		
High LDL-C	Naïve	12	6,725	4.7 (2.2–8.2)	168.9	92.1%	<0.01	37,398.3	<0.01
	ART	16	14,585	14.6 (9.7–20.3)	1,301.7	98.8%	<0.01		
Low HDL-C	Naïve	10	5,979	36.8 (21.5–53.5)	419.8	97.9%	<0.01	1,931.0	<0.01
	ART	16	14,417	30.0 (22.8–37.7)	902.5	98.3%	<0.01		

### 3.3 Difference of dyslipidemia and serum lipids among PLWH

**Figure 5:**
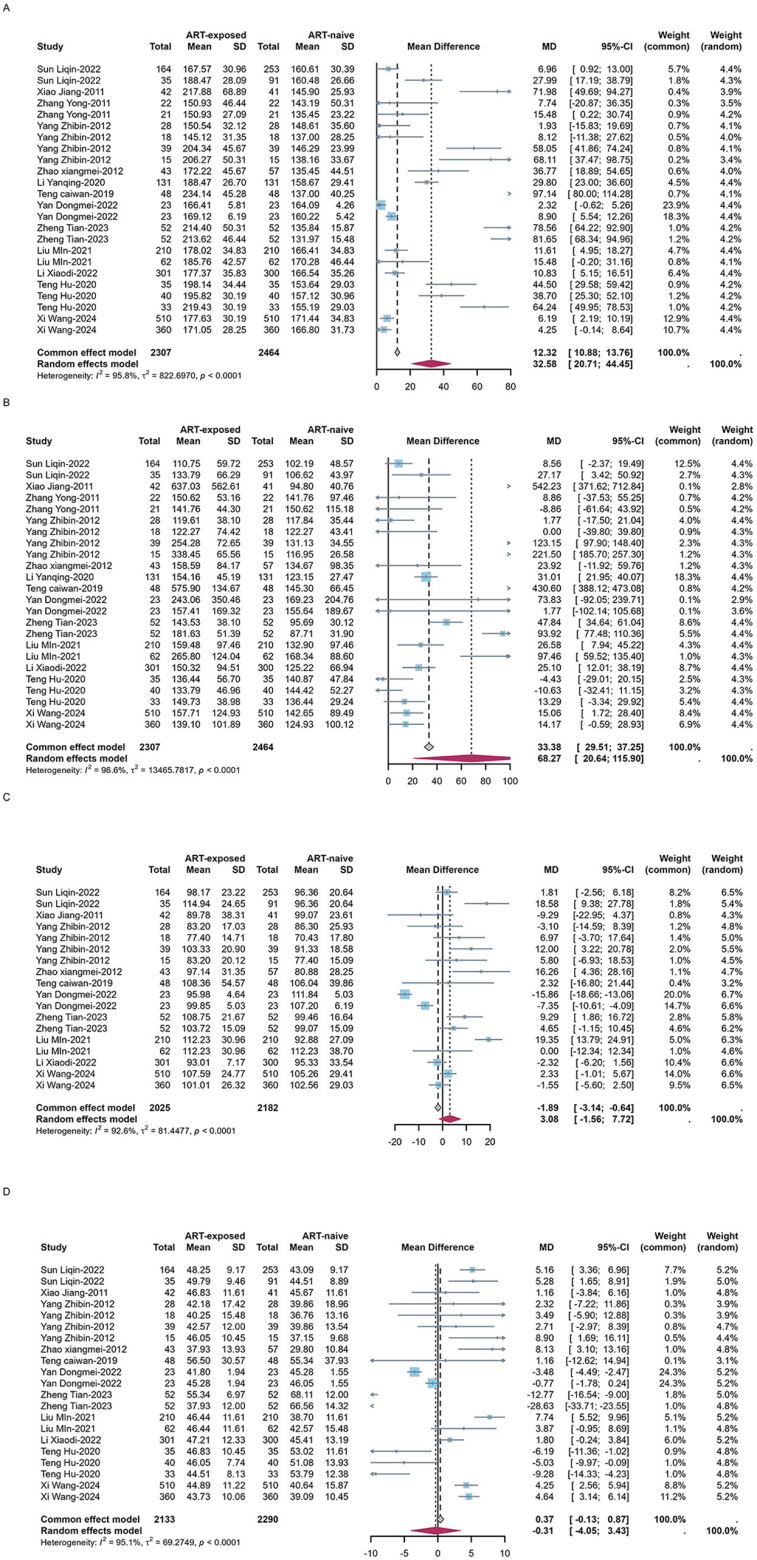
Forest plots of the pooled MDs of serum TC **(A)**, TG **(B)**, LDL-C **(C)** and HDL-C **(D)** among PLWH.

High TG and low HDL-C were the most prevalent forms of dyslipidemia observed in both ART-naïve and experienced PLWH in China. Among ART-naïve individuals, the estimated prevalence of high TC, high TG, high LDL-C, and low HDL-C was 11.1% (95%CI: 8.3–15.0%), 22.6% (95%CI: 16.3–29.4%), 4.7% (95%CI: 2.2%–8.2%), and 36.8% (95%CI: 21.5–53.5%). In contrast, the prevalence was significant higher for high TC (estimated: 23.5%, 95%CI: 15.8–32.2%), high TG (estimated: 40.6%, 95%CI: 31.6–49.9%) and high LDL-C (estimated: 14.6%, 95%CI: 9.7–20.3%) while low HDL-C prevalence was 30.0% (95%CI: 22.8–37.7%) among ART-experienced PLWH in China (Table 1 and Supplementary Figure S1). The serum concentration of TC (estimated MD = 32.6 mg/dl, 95%CI: 20.7–44.5 mg/dl), TG (estimated MD = 68.3 mg/dl, 95%CI: 20.6–115.9 mg/dl) and LDL-C (estimated MD = 3.1 mg/dl, 95%CI:−1.6 to 7.7 mg/dl) was elevated after initiating ART while HDL-C (estimated MD = −0.3 mg/dl, 95%CI:−4.1 to 3.4 mg/dl) was decreased (Figure 5).

### 3.4 Factors associated with overall dyslipidemia in ART-experienced PLWH

The prevalence of dyslipidemia in ART-experienced PLWH exhibited significant variations across region, BMI group, baseline CD4 + T-cell count, and TCM therapy (*p* for comparison <0.05) (Table 2). The prevalence of dyslipidemia in South China was higher than that in North China (59.8% vs. 45.0%, Q_B_ = 4.1, *P* = 0.04). Individuals with a BMI ≥24 kg/m^2^ had a significant elevated prevalence relative to those with a lower BMI (63.5% vs. 49.8%, Q_B_ = 6.0, *P* = 0.049). Notably, PLWH with a baseline CD4+ T-cell count over 500 cells/μl displayed a higher dyslipidemia prevalence compared with those with lower counts (35.5% vs. 23.3%, Q_B_ = 8.0, *P* = 0.02). Individuals receiving TCM therapy had a higher prevalence of dyslipidemia compared to non-recipients (34.0% vs. 23.7%, Q_B_ = 6.2, *P* = 0.01) (Table 2).

**Table 2:** Subgroup meta-analysis for prevalence of dyslipidemia among ART-experienced PLWH.

Subgroups	No. of study	Sample size	Pooled prevalence (%) （95%CI）	Heterogeneity		Meta regression		Q_B_	*p* for comparison
				*I* ^2^	*p*	*R* ^2^	*p*		
Region	30	28,388				7.7%	0.07	4.1	0.04*
North	19	24,386	45.0 (36.8–55.0)	98.2%	<0.01				
South	11	4,002	59.8 (49.6–72.2)	99.6%	<0.01				
Design	31	31,706				0.0%	0.41	0.7	0.41
Cross-sectional	16	19,160	48.5 (39.5–59.6)	99.4%	<0.01				
Cohort	15	12,546	54.9 (44.6–67.6)	99.5%	<0.01				
Age	12	10,736				0.0%	0.65	0.2	0.65
<50	6	6,544	31.8 (20.2–50.1)	99.50%	<0.01				
≥50	6	4,192	36.8 (24.1–56.2)	99.3%	<0.01				
Gender	24	21,552				0.0%	0.63	0.2	0.63
Male	12	13,528	39.3 (30.3–50.9)	99.3%	<0.01				
Female	12	8,024	36.1 (28.8–45.1)	98.2%	<0.01				
BMI	15	10,196							
<18.5 kg/m^2^	5	1,177	36.5 (24.8–53.8)	94.2%	<0.01	27.6%	0.03	6.0	0.049*
18.5–23.9 kg/m^2^	5	6,471	49.8 (38.0–65.4)	99.2%	<0.01				
≥24 kg/m^2^	5	2,548	63.5 (50.3–80.3)	97.7%	<0.01				
Duration of ART	17	6,809				0.0%	0.66	0.2	0.64
<5 years	12	4,410	38.8 (27.4–54.8)	99.0%	<0.01				
≥5 years	5	2,399	44.7 (27.5–72.5)	99.5%	<0.01				
Baseline CD4 counts	6	4,972				48.2%	0.049	8.0	0.02*
≤350 cells/μl	2	2,160	23.3 (21.3–25.5)	24.0%	0.25				
350–500 cells/μl	2	1,136	27.9 (20.8–37.4)	88.0%	<0.01				
>500 cells/μl	2	1,676	35.5 (26.5–48.2)	93.0%	<0.01				
Fasting blood glucose	12	6,638				9.7%	0.14	2.1	0.14
<6.1 mmol/L	6	5,587	38.4 (26.9–54.9)	99.2%	<0.01				
≥6.1 mmol/L	6	751	55.9 (39.3–79.5)	96.0%	<0.01				
TCM Therapy	10	9,067				36.3%	0.02	6.2	0.01*
No	5	7,717	24.7 (21.0–29.12)	95.8%	<0.01				
Yes	5	1,350	34.0 (28.1–41.1)	80.3%	<0.01				

QB, Cochrane Q test for heterogeneity between groups.

ART, antiretroviral treatment; TCM Therapy, traditional Chinese medicine.

* *p* ≤ 0.05.

### 3.5 Effects of ART regimens on dyslipidemia and serum lipids

No statistically significant intergroup differences were observed for the prevalence of high TC, high TG, or high LDL-C. LPV/r-based regimens were associated with highest prevalence of high TG (51.2%), followed by INSTI-based regimens (44.8%) and EFV-based regimens (38.4%), though these differences did not reach statistical significance (Q_B_ = 0.52, *P* = 0.77). The estimated prevalence of low HDL-C among ART-experienced PLWH revealed significant difference between three regimens (Q_B_ = 18.24, *P* < 0.01), with INSTI-based regimens showing the highest prevalence (24.0%), significantly exceeding both EFV-based (15.0%) and LPV/r-based regimens (10.9%) (Table 3 and Supplementary Figure S2). INSTI-based regimens were associated with highest mean difference of serum HDL-C (4.4 mg/dl, Q_B_ = 6.11, *P* = 0.047), compared to EFV-based regimens (0.06 mg/dl) and LPV/r-based regimens (−11.3 mg/dl) (Table 4 and Supplementary Figure S3).

**Table 3:** Effects of LPV/r-based regimens and EFV-based regimens on dyslipidemia.

Regimens	No. of study	Sample size	Pooled prevalence (%) （95%CI）	Heterogeneity		Meta-regression		Q_B_	*p* for comparison
				*I* ^2^	*p*	*R* ^2^	*p*		
Overall Dyslipidaemias	10	2,701				0.0%	0.31	1.49	0.47
EFV-based	5	1,903	54.8 (36.4–82.7)	94.1%	<0.01				
LPV/r-based	3	209	67.4 (44.9–100)	87.6%	<0.01				
INSTI-based	2	589	71.7 (62.2–82.7)	65.5%	0.09				
High TC	11	4,190				0.0%	0.32	1.51	0.47
EFV-based	5	3,249	25.1 (16.4–38.5)	95.9%	<0.01				
LPV/r-based	3	481	28.7 (16.8–49.3)	90.4%	<0.01				
INSTI-based	3	460	21.1 (17.6–25.2)	31.5%	0.23				
High TG	13	4,462				0.0%	0.78	0.52	0.77
EFV-based	6	3,459	38.4 (23.4–62.9)	98.0%	<0.01				
LPV/r-based	4	543	51.2 (25.9–100)	89.4%	<0.01				
INSTI-based	3	460	44.8 (40.5–49.7)	62.4%	0.07				
High LDL-C	7	3,554				14.9%	0.16	5.02	0.08
EFV-based	2	2,665	27.8 (18.8–40.9)	97.5%	<0.01				
LPV/r-based	2	429	25.9 (22.1–30.4)	0.0%	0.91				
INSTI-based	3	460	43.7 (28.4–67.1)	95.1%	<0.01				
Low HDL-C	7	3,554				84.4%	<0.01	18.24	<0.01*
EFV-based	2	2,665	15.0 (12.9–17.5)	64.6%	0.09				
LPV/r-based	2	429	10.9 (4.4–27.0)	80.6%	0.02				
INSTI-based	3	460	24.0 (20.4–28.2)	0.0%	0.88				

QB, Cochrane Q test for heterogeneity between groups.

TC, total cholesteroll; TG, triglycerides.

* *p* ≤ 0.05.

**Table 4:** Effects of LPV/r-based regimens and EFV-based regimens on MDs of serum lipids.

Regimens	No. of study	Sample size	Mean difference (mg/dl) (95%CI)	Heterogeneity		Meta-regression		QB	*p* for comparison
				*I* ^2^	*p*	*R* ^2^	*p*		
TC	17	1,820				0.5%	0.42	1.64	0.44
EFV-based	11	1,050	24.8 (9.4–40.2)	94.1%	<0.01				
LPV/r-based	4	169	43.4 (8.0–78.8)	94.2%	<0.01				
INSTI-based	2	601	16.5 (−4.8 to 37.8)	92.7%	<0.01				
TG	17	1,820				0.0%	0.78	2.39	0.3
EFV-based	11	1,050	32.2 (−9.6 to 74.0)	94.2%	<0.01				
LPV/r-based	4	169	54.1 (6.4–101.9)	94.5%	<0.01				
INSTI-based	2	601	18.0 (6.3–29.6)	0.0%	0.38				
LDL-C	12	1,669				0.0%	0.65	0.79	0.67
EFV-based	8	954	1.9 (−6.0–9.7)	95.8%	<0.01				
LPV/r-based	2	114	3.8 (−1.4 to 9.1)	0.0%	0.50				
INSTI-based	2	601	9.9 (−6.0 to 25.7)	90.6%	<0.01				
HDL-C	15	1,777				17.9%	0.09	6.11	0.047*
EFV-based	10	1,029	0.06 (−4.3 to 4.3)	96.0%	<0.01				
LPV/r-based	3	147	−11.3 (−29.8 to 7.17)	97.6%	<0.01				
INSTI-based	2	601	4.4 (2.9–6.0)	0.0%	0.61				

QB, Cochrane Q test for heterogeneity between groups,.

TC, total cholesterol; TG, triglycerides; LDL-C, low-density lipoprotein cholesterol; HDL-C, high-density lipoprotein cholesterol.

* *p* ≤ 0.05.

### 3.6 Publication bias and sensitivity analysis

We investigated the existence of publication bias using the Egger's test and funnel plots. Most results of both methods (Funnel plots and Egger's tests: Supplementary Figures S4–S6) indicated no publication bias. However, the application of the trim-and-fill method significantly altered the estimated prevalence of high LDL-C among ART-naïve PLWH (5.0% to 9.2%), emphasizing the importance of considering publication bias in meta-analyses (Supplementary Figures S5, S6). The results of sensitivity analysis also showed that the results were relatively stable (Supplementary Figures S8–S10).

## 4 Discussion

The overall estimated prevalence of dyslipidemia in ART-experienced PLWH was 55.1%, lower than reported in two cohort studies (75.6% and 84.3%) but similar to a global meta-analysis (53%) (33–35). The prevalence of dyslipidemia among the general Chinese adult population was 35.6%, which was obviously lower than that in Chinese PLWH before and after initiating ART (36). Immune activation and inflammation were stimulated by HIV (37–39), and activated monocytes were recruited by inflammatory cytokine to the vascular endothelium where it absorbed cholesterol (10), resulting the increased serum concentrations of cholesterols and dysfunction of HDL.

The most common dyslipidemia in our study were low HDL-C among ART-naïve individuals, possibly due to the HIV infection-related impairment to HDL-C (9, 40). A long-term cohort study in United States showed that notable declines in serum levels of TC, HDL-C, and LDL-C were observed in people newly infected with HIV, while levels of TC and LDL-C have been observed to increase despite HDL-C levels continued to remain low after ART, which was similar in Chinese PLWH (41, 42). Our study also verified the same change in consistence with a meta-analysis focused on lipid profiles in PLWH (43).

Living in South China, BMI over 24, high CD4T-cell count at baseline and taking TCM therapy were identified as risk factors for dyslipidemia in our study. In northern China, wheat-based products, which are considered whole grains and rich in dietary fiber, are the dietary staple. These foods have a relatively low impact on blood glucose metabolism. In contrast, southern China predominantly consumes refined rice as a staple, which has less dietary fiber and a faster blood sugar response (44, 45). Long-term southern diet may contribute to a higher risk of insulin resistance and subsequent dyslipidemia. Overweight and obesity have been closely linked to dyslipidemia (46), and a recent mendelian analysis revealed a causal association between higher BMI, elevated TG, and reduced HDL-C, which was observed in PLWH and our study (47, 48). Additionally, CD4+ T-cell count over 500 cells/μl at baseline was related with higher prevalence of dyslipidemia among PLWH in this meta-analysis, possibly due to the limited number of studies reporting dyslipidemia prevalence across different baseline CD4+ T-cell count subgroups. Our analysis also suggests that taking traditional Chinese medicine alongside antiretroviral therapy may increase the risk of dyslipidemia in PLWH, though this was not supported by a study in Taiwan, possibly due to limited studies (49). The studies reporting on TCM as an additional therapy were exclusively based on patients from Henan province who participated in the National Chinese Medicine Treatment AIDS Trial Program. PLWH in this project would take *Yi Ai Kang* capsules, a Chinese patent drug specifically used for treating HIV infection for free. While TCM adjuvant therapy can mitigate adverse side effects of antiviral drugs in HIV/AIDS patients, it may increase hepatic metabolic load, thereby disrupting lipid metabolism and potentially leading to dyslipidemia (50).

PLWH generally need to take lifelong administration of antiretroviral drugs to suppress HIV virus replication and rebuild their immune function, while long-term ART leads to a variety of toxicities, particularly dyslipidemia. Our results supported that long term of ART was associated with the higher prevalence of dyslipidemia, which was similar to researches in the United States and Cameroon (41, 51, 52).

Antiretroviral therapies are well-known to influence lipid profiles in PLWH. Our finding supported that INSTI-based regimens showed the greatest increase in HDL-C. However, despite the highest HDL-C elevation in the INSTI-based regimens, it still exhibited the highest post-treatment HDL-C abnormality rate. This apparent discrepancy may stem from differences in observation periods—most INSTI-related studies only reported first-year data, lacking longer-term follow-up. In fact, prevalence of low HDL-C tend to improve as ART duration prolonging (53, 54). Thus, the high prevalence of low HDL-C in INSTI-based regimens likely reflects its shorter observation window (only 12 months), whereas other regimens, with longer follow-up, demonstrate lower prevalence due to gradual improvement. Our study also find that LPV/r-based regimens were associated with the greatest increases in serum TC and TG levels, along with the highest rates of high TC and high TG among all regimens, although these differences did not reach statistical significance. These observations align with two Spanish cohort studies (55, 56).

Accurately assessment of dyslipidemia among PLWH is essential for developing effective prevention, monitoring, and management strategies. This study provides four contributions: (1) establishing comprehensive national estimates of dyslipidemia among PLWH in China; (2) characterizing temporal changes in the prevalence of specific lipid abnormalities and lipid changes before and after ART; (3) identifying clinically relevant risk factors for dyslipidemia in ART-experienced PLWH; and (4) conducting a comparative effectiveness analysis of three widely used antiretroviral regimens in China.

There were obvious limitations in our study. First, most of the included studies were conducted in Beijing and Henan (18/44), limiting the national representativeness of the findings. Besides, publication bias in high LDL-C prevalence before ART significantly skewed the pooled results (initial and corrected estimates: 5.0%–9.2%). This suggests that studies may underestimate the prevalence of elevated LDL-C, a key cardiovascular risk factor. For prevalence analysis, we excluded studies with small sample sizes (<100) to avoid small study effect, while the meta-analysis of mean differences excluded participants with diabetes, hypertension, and heart disease, focusing on healthier individuals and possibly not reflecting the broader HIV-positive population on ART. This discrepancy between the estimated prevalence and real-world prevalence of dyslipidemia should be interpreted cautiously. Additionally, we were unable to distinguish between PLWH in stable condition and those with uncontrolled viremia due to the lack of data on HIV viral suppression at baseline and during follow-up. Furthermore, potential confounders such as the use of lipid-lowering drugs and levels of physical activity, which can significantly affect lipid profiles, were not reported in most studies. A nationwide study by the China National Survey of Chronic Kidney Disease Working Group found 34.0% of Chinese adults had dyslipidemia, with low awareness (31.0%), treatment (19.5%), and control rates (8.9%) (57). In a cross-sectional study of 973 ART-naïve HIV patients across 11 Chinese provinces, none of the 30 patients qualifying for lipid-lowering therapy had received statins (58). China demonstrates high dyslipidemia prevalence but poor management, with HIV-affected populations showing particularly severe care gaps. Lastly, this study did not collect longitudinal data on body weight or BMI changes in PLWH, thus limiting our ability to assess the potential impact of INSTI-associated weight gain on lipid abnormalities, despite controlling for chronic diseases and lipid-lowering medication use to clarify the influence of treatment regimens. Previous studies have suggested that INSTI may indirectly affect lipid metabolism by promoting weight gain, and this confounding factor may partially explain the observed association between INSTI-based regimens and dyslipidemia in our study (59).

## 5 Conclusion

In summary, dyslipidemia prevalence was high among PLWH in China, both before and after ART initiation. The most common dyslipidemias were elevated triglycerides and low HDL-C levels. INSTI-based regimens were associated with highest prevalence of low HDL-C in early stage of ART and LPV/r-based regimens had a stronger impact on increasing TC and TG levels compared to other regimens. Regular lipid monitoring is essential for Chinese PLWH before and after imitating ART. Early intervention, such as switching ART regimens, using lipid-lowering drugs, promoting exercise were should be prioritized for PLWH with dyslipidemia.

## Data Availability

The original contributions presented in the study are included in the article/Supplementary Material, further inquiries can be directed to the corresponding authors.
