# Supplementary figures and images for: The prevalence of dyslipidemia and its correlation with anti-retroviral therapy among people living with HIV in China: a systematic review and meta-analysis
Source: Front Cardiovasc Med. 2025 Jun 13;12:1498165. doi: 10.3389/fcvm.2025.1498165 (PMC12202347; doi:10.3389/fcvm.2025.1498165)

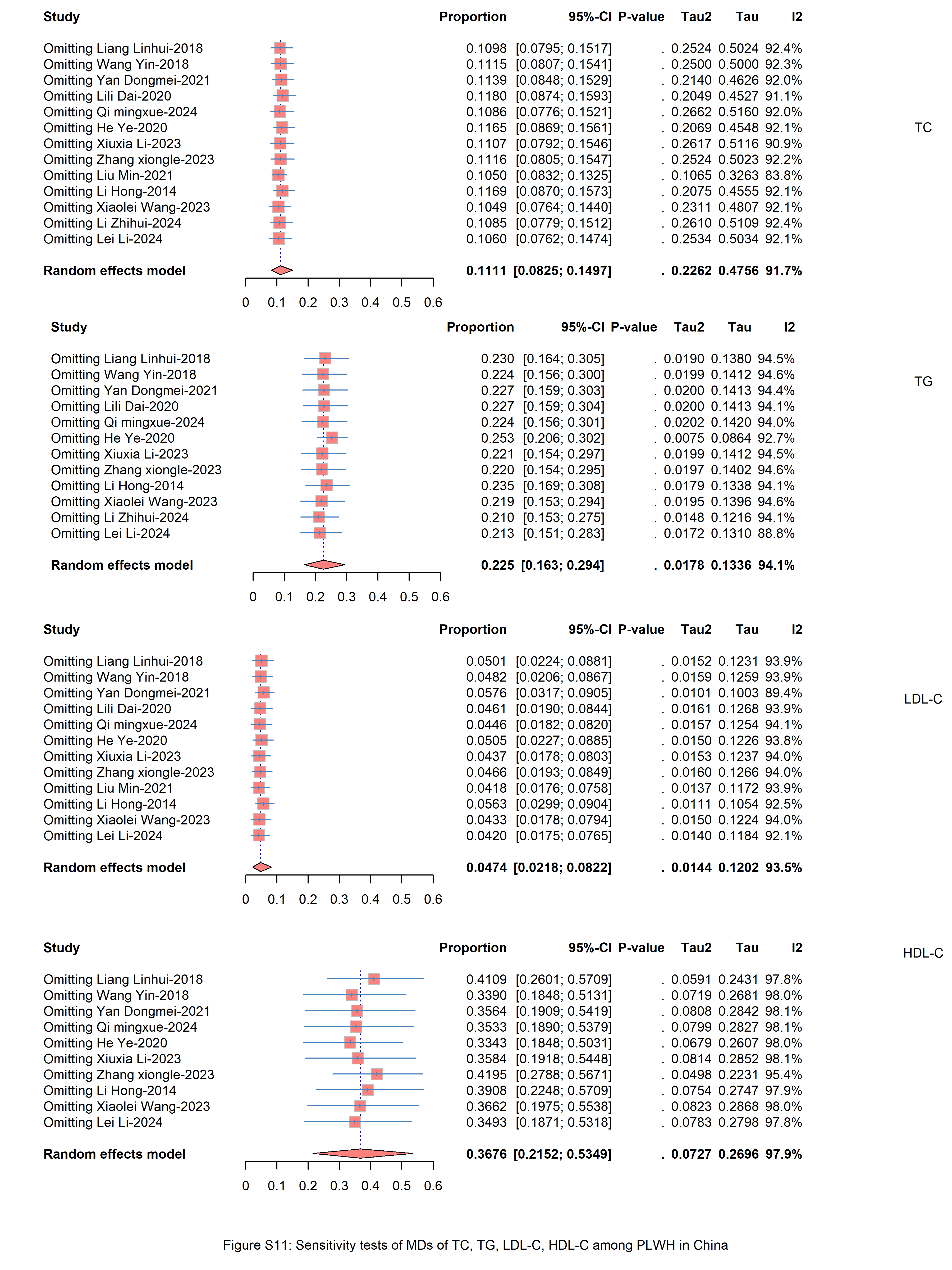

Supplement: Supplementary file 1 [file Datasheet1.zip › Supplementary Figures/Figure S10.Sensitivity tests of MDs of TC, TG, LDL-C, HDL-C among PLWH in China..tiff]

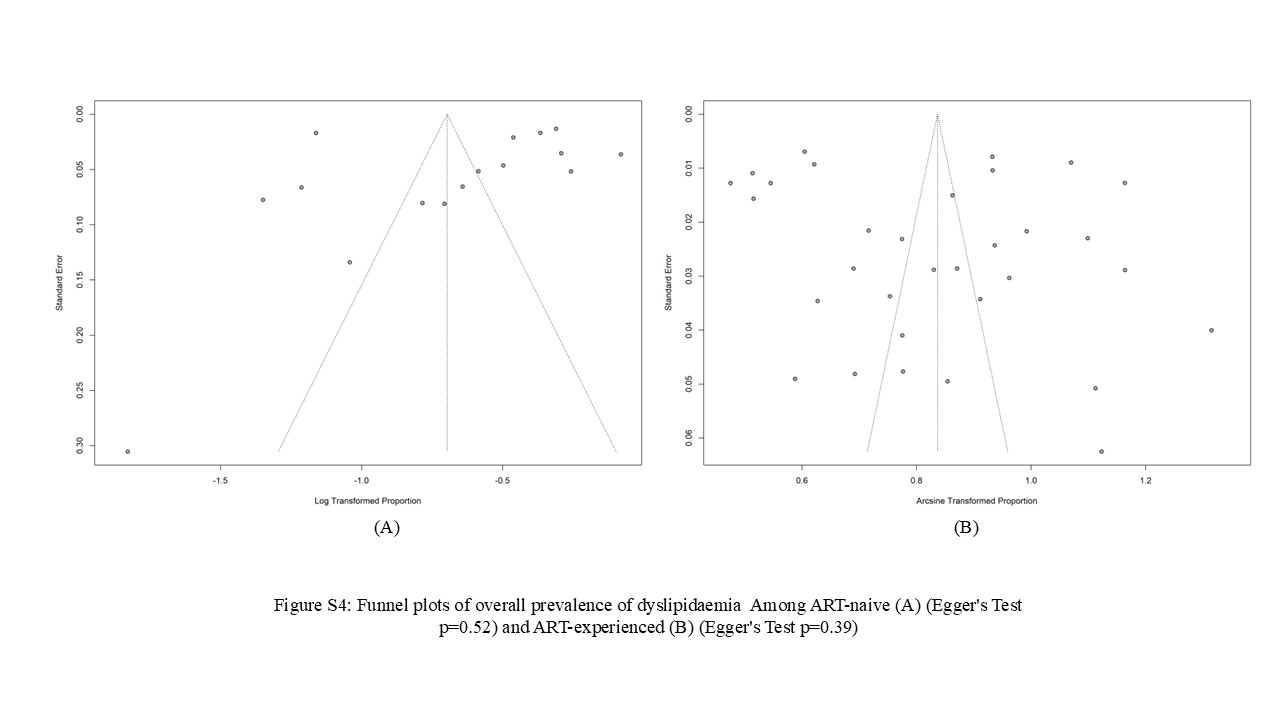

Supplement: Supplementary file 1 [file Datasheet1.zip › Supplementary Figures/Figure S4 funnel plots of overall prevalence of dyslipidemias among ART-naïve (A) (Egger’s Test p=0.52) and ART-experienced(B) (Egger’s Test p=039).jpg]

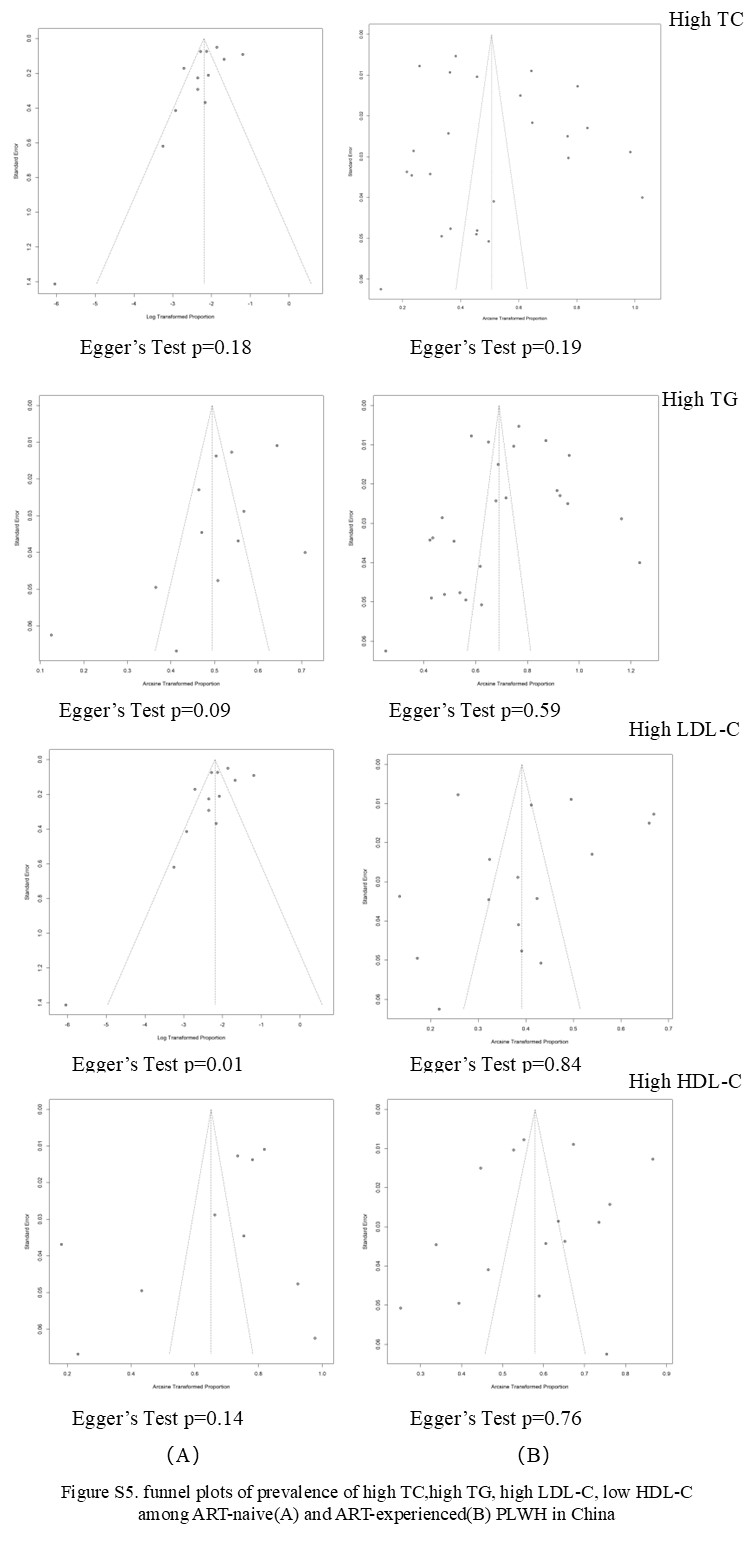

Supplement: Supplementary file 1 [file Datasheet1.zip › Supplementary Figures/Figure S5. funnel plots of prevalence of high TC, high TG, high LDL-C and low HDL-C among ART-naïve (A) and ART-experienced (B) PLWH in China.jpg]

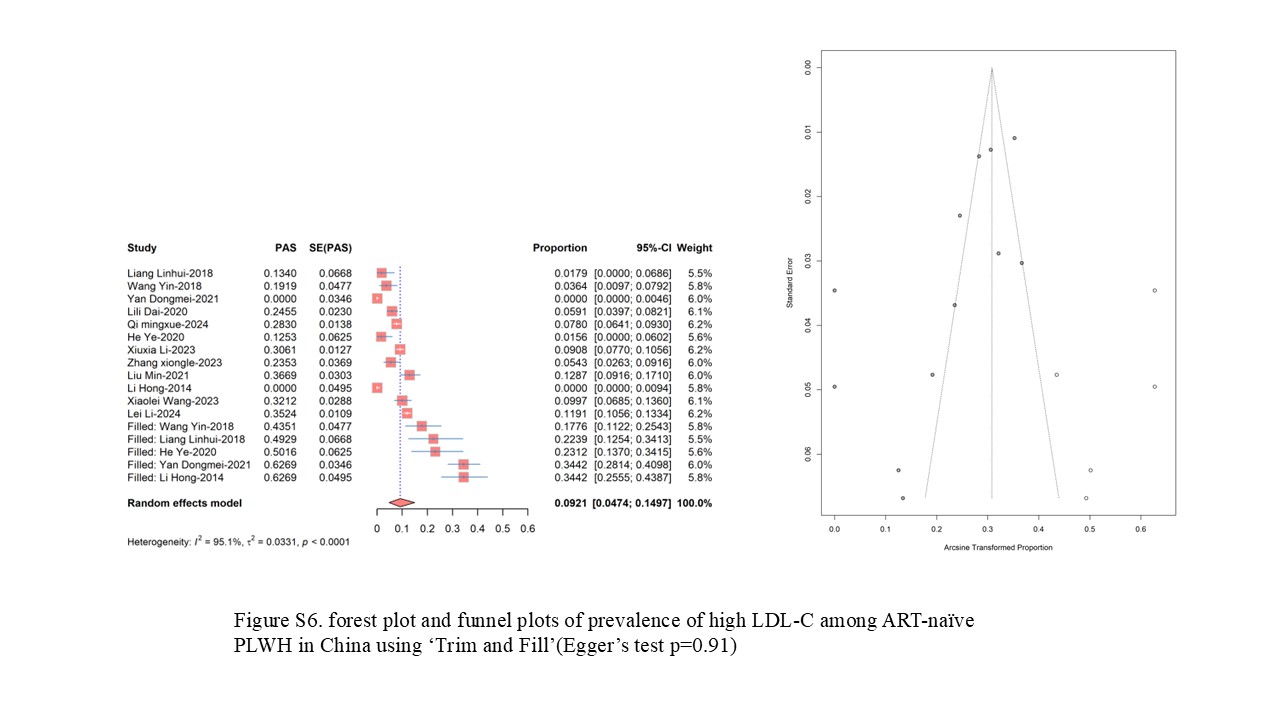

Supplement: Supplementary file 1 [file Datasheet1.zip › Supplementary Figures/Figure S6. forest plot and funnel plots of prevalence of high LDL-C among ART-naïve PLWH in China using ‘Trim and Fill’(Egger’s test p=0.91).jpg]

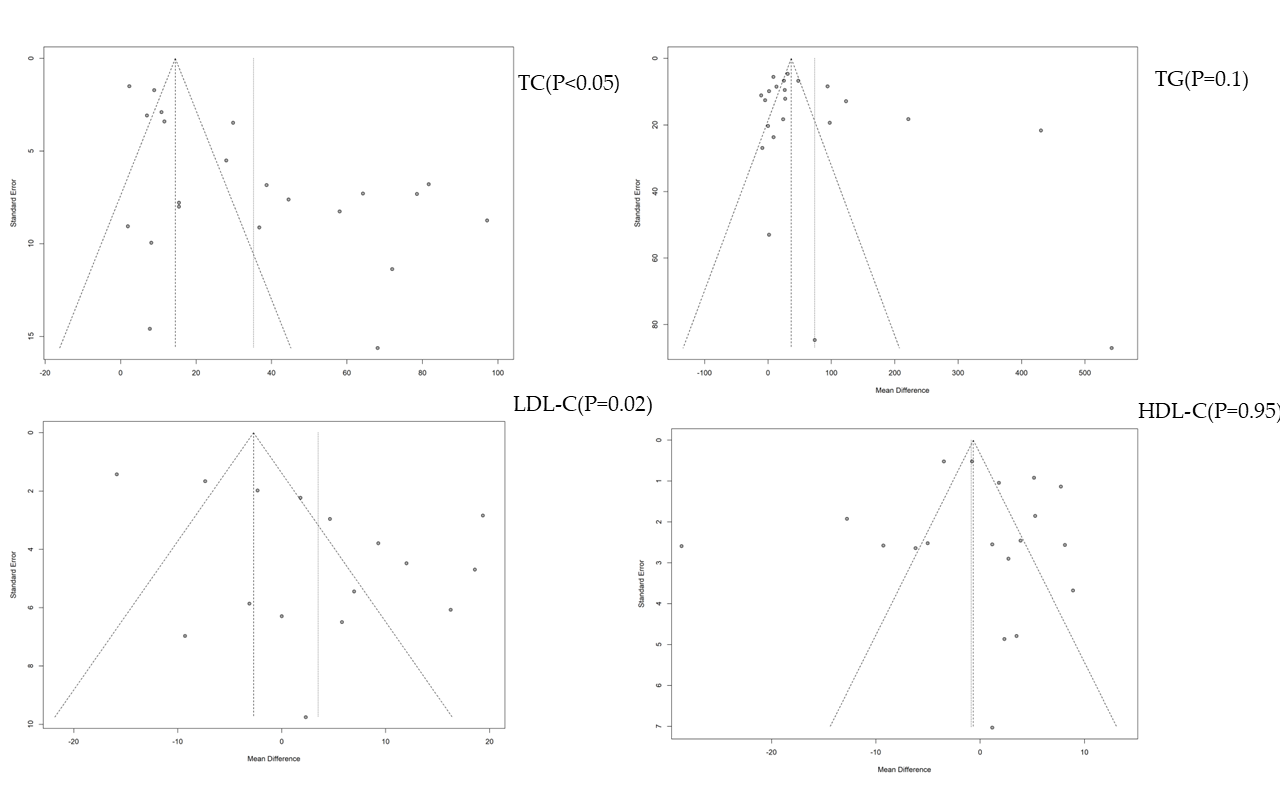

Supplement: Supplementary file 1 [file Datasheet1.zip › Supplementary Figures/Figure S7. funnel plots of MDs of TC, TG, LDL-C, HDL-C among PLWH in China.TIF]

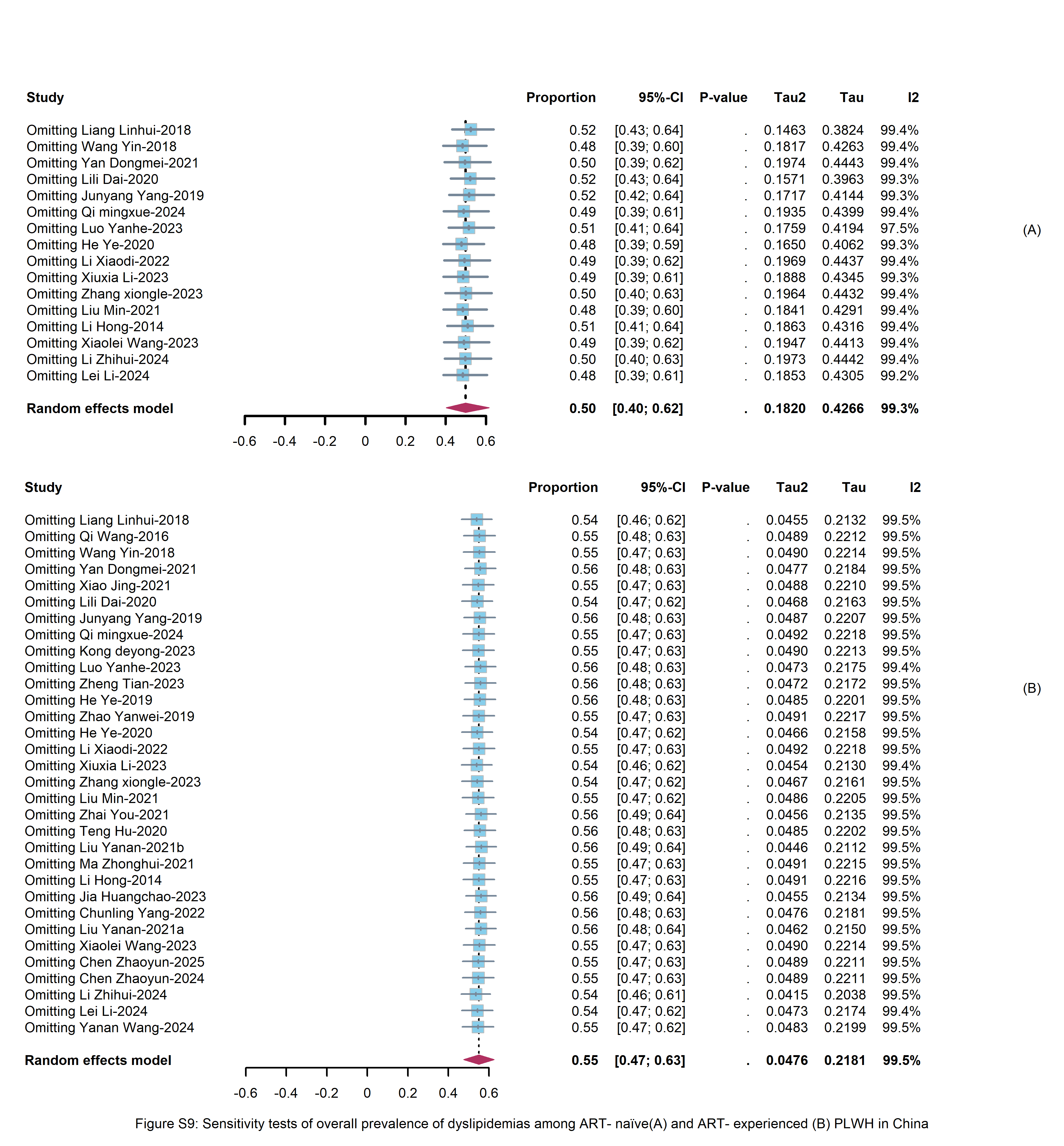

Supplement: Supplementary file 1 [file Datasheet1.zip › Supplementary Figures/Figure S8. Sensitivity test of overall prevalence of dyslipidemias among ART- naïve(A) and ART- experienced (B) PLWH in China;.tiff]
