# Supplementary material for: The prevalence of dyslipidemia and its correlation with anti-retroviral therapy among people living with HIV in China: a systematic review and meta-analysis
Source: Front Cardiovasc Med. 2025 Jun 13;12:1498165. doi: 10.3389/fcvm.2025.1498165 (PMC12202347; doi:10.3389/fcvm.2025.1498165)
Supplement: Supplementary file 1 [file Datasheet1.zip › Supplementary Tables.zip!/Table S2 PubMed Search Strategy.docx]

**Table S2** PubMed Search Strategy

Search strategies in PubMed were used in other English databases equally.

| Database:PubMed  Search Strategy: | |
| --- | --- |
| Search | Keywords |
| #1 | ((((((((((((((((((((((((("HIV"[Mesh]) OR (Human Immunodeficiency Virus[Title/Abstract])OR (Immunodeficiency Virus, Human[Title/Abstract])) OR (Immunodeficiency Viruses, Human[Title/Abstract])) OR (Virus, Human Immunodeficiency[Title/Abstract])) OR (Human Immunodeficiency Viruses[Title/Abstract])) OR ( Human T Cell Lymphotropic Virus Type III[Title/Abstract])) OR (Human T-Cell Lymphotropic Virus Type III[Title/Abstract])) OR (Human T-Cell Leukemia Virus Type III[Title/Abstract])) OR (Human T Cell Leukemia Virus Type III[Title/Abstract])) OR (LAV-HTLV-III[Title/Abstract])) OR (Lymphadenopathy-Associated Virus[Title/Abstract])) OR (Lymphadenopathy Associated Virus[Title/Abstract])) OR (Lymphadenopathy-Associated Viruses[Title/Abstract])) OR (Virus, Lymphadenopathy-Associated[Title/Abstract])) OR (Viruses, Lymphadenopathy-Associated[Title/Abstract])) OR (Human T Lymphotropic Virus Type III[Title/Abstract])) OR (Human T-Lymphotropic Virus Type III[Title/Abstract])) OR (AIDS Virus[Title/Abstract])) OR (AIDS Viruses[Title/Abstract])) OR (Virus, AIDS[Title/Abstract])) OR (Viruses, AIDS[Title/Abstract])) OR ( Acquired Immune Deficiency Syndrome Virus[Title/Abstract])) OR (Acquired Immunodeficiency Syndrome Virus[Title/Abstract])) OR (HTLV-III[Title/Abstract])) |
| #2 | ((((((((Highly Active Antiretroviral Therapy[MeSH Terms]) OR (Antiretroviral Therapy, Highly Active[Title/Abstract])) OR (ART[Title/Abstract])) OR (Combination Antiretroviral Therapy[Title/Abstract])) OR (Antiretroviral Therapies, Combination[Title/Abstract])) OR (Combination Antiretroviral Therapies[Title/Abstract])) OR (Therapies, Combination Antiretroviral[Title/Abstract])) OR (Therapy, Combination Antiretroviral[Title/Abstract])) OR ((((((HIV Protease Inhibitors[MeSH Terms]) OR (Inhibitors, HIV Protease[Title/Abstract])) OR (Protease Inhibitors, HIV[Title/Abstract])) OR (HIV Protease Inhibitor[Title/Abstract])) OR (Inhibitor, HIV Protease[Title/Abstract])) OR (Protease Inhibitor, HIV[Title/Abstract])) |
| #3 | (((("Dyslipidemias"[Mesh]) OR (Dyslipidemia[Title/Abstract])) OR (Dyslipoproteinemias[Title/Abstract])) OR (Dyslipoproteinemia[Title/Abstract])) |
| #4 | (((((((("Hyperlipidemias"[Mesh]) OR (Hyperlipemia[Title/Abstract])) OR (Hyperlipemias[Title/Abstract])) OR (Hyperlipidemia[Title/Abstract])) OR (Lipidemia[Title/Abstract])) OR (Lipidemias[Title/Abstract])) OR (Lipemia[Title/Abstract])) OR (Lipemias[Title/Abstract])) |
| #5 | (((((((((((((hypercholesterolemia[MeSH Terms]) OR (Hypercholesterolemias[Title/Abstract])) OR (High Cholesterol Levels[Title/Abstract])) OR (Cholesterol Level, High[Title/Abstract])) OR (Cholesterol Levels, High[Title/Abstract])) OR (High Cholesterol Level[Title/Abstract])) OR (Level, High Cholesterol[Title/Abstract])) OR (Levels, High Cholesterol[Title/Abstract])) OR (Elevated Cholesterol[Title/Abstract])) OR (Cholesterol, Elevated[Title/Abstract])) OR (Cholesterols, Elevated[Title/Abstract])) OR (Elevated Cholesterols[Title/Abstract])) OR (Hypercholesteremia[Title/Abstract])) OR (Hypercholesteremias[Title/Abstract]) |
| #6 | (hypertriglyceridemia[MeSH Terms]) OR (Hypertriglyceridemias[Title/Abstract]) |
| #7 | (total cholesterol[MeSH Terms]) OR (Epicholesterol[Title/Abstract]) |
| #8 | (((triglycerides[MeSH Terms]) OR (Triacylglycerols[Title/Abstract])) |
| #9 | (((((((((((Cholesterol, HDL[MeSH Terms]) OR (alpha-Lipoprotein Cholesterol[Title/Abstract])) OR (Cholesterol, alpha-Lipoprotein[Title/Abstract])) OR (alpha Lipoprotein Cholesterol[Title/Abstract])) OR (HDL Cholesterol[Title/Abstract])) OR (High Density Lipoprotein Cholesterol[Title/Abstract])) OR (Cholesterol, HDL2[Title/Abstract])) OR (HDL2 Cholesterol[Title/Abstract])) OR (HDL(2) Cholesterol[Title/Abstract])) OR (Cholesterol, HDL3[Title/Abstract])) OR (HDL3 Cholesterol[Title/Abstract])) OR (HDL(3) Cholesterol[Title/Abstract] |
| #10 | (((((((Cholesterol, LDL[MeSH Terms]) OR (Low Density Lipoprotein Cholesterol[Title/Abstract])) OR (beta-Lipoprotein Cholesterol[Title/Abstract])) OR (Cholesterol, beta-Lipoprotein[Title/Abstract])) OR (beta Lipoprotein Cholesterol[Title/Abstract])) OR (LDL Cholesterol[Title/Abstract])) OR (Cholesteryl Linoleate, LDL[Title/Abstract])) OR (LDL Cholesteryl Linoleate[Title/Abstract]) |
| #11 | (China[Title/Abstract]) OR (Chinese[Title/Abstract]) |
| #12 | #1 AND #2 AND #11 AND #3 |
| #13 | #1 AND #2 AND #11 AND #4 |
| #14 | #1 AND #2 AND #11 AND #5 |
| #15 | #1 AND #2 AND #11 AND #6 |
| #16 | #1 AND #2 AND #11 AND #7 |
| #17 | #1 AND #2 AND #11 AND #8 |
| #18 | #1 AND #2 AND #11 AND #9 |
| #19 | #1 AND #2 AND #11 AND #10 |

AND ((“1949/10/01”[Date - Publication] : "2025/03/01"[Date - Publication]))
