# Supplementary material for: The prevalence of dyslipidemia and its correlation with anti-retroviral therapy among people living with HIV in China: a systematic review and meta-analysis
Source: Front Cardiovasc Med. 2025 Jun 13;12:1498165. doi: 10.3389/fcvm.2025.1498165 (PMC12202347; doi:10.3389/fcvm.2025.1498165)
Supplement: Supplementary file 1 [file Datasheet1.zip › Supplementary Tables.zip!/Table S3 CNKI search strategy.docx]

**Table S3** CNKI search strategy

Search strategies in CNKI were used in other Chinese databases equally.

| Database: Chinese Biomedical Literature Database  Search Strategy: | |
| --- | --- |
| Search | Keywords |
| #1 | (主题：HIV(精确））OR（篇关摘：HIV+人类免疫缺陷病毒（精确））OR（主题：AIDS（精确））OR（篇关摘：AIDS+艾滋病(精确）） |
| #2 | (主题：ART （精确））OR（篇关摘：ART+高效抗逆转录病毒治疗（精确））） |
| #3 | （结果中检索#2） |
| #4 | #3 AND （（篇关摘：脂质（精确）） OR （篇关摘：胆固醇（精确））） |
| #5 | #3 AND （（篇关摘：血脂异常（精确）） OR （篇关摘：血脂异常+高脂血症 血脂异常+高脂蛋白血症（精确））） |
| #6 | #3 AND （（篇关摘：血脂异常（精确）） OR （篇关摘：胆固醇，LDL+低密度脂蛋白胆固醇（精确））） |
| #7 | #3 AND （（篇关摘：胆固醇,HDL（精确）） OR （篇关摘：胆固醇,HDL+高密度脂蛋白胆固醇（精确））） |
| #8 | #3 AND （（篇关摘：TC+脂质+血脂（精确）） OR （篇关摘：TC+血清总胆固醇（精确））） |
| #9 | #3 AND （（篇关摘：高甘油三酯血症（精确）） OR （篇关摘：TG+血清甘油三酯（精确））） |
