# Supplementary material for: The prevalence of dyslipidemia and its correlation with anti-retroviral therapy among people living with HIV in China: a systematic review and meta-analysis
Source: Front Cardiovasc Med. 2025 Jun 13;12:1498165. doi: 10.3389/fcvm.2025.1498165 (PMC12202347; doi:10.3389/fcvm.2025.1498165)
Supplement: Supplementary file 1 [file Datasheet1.zip › Supplementary Tables.zip!/Table S4 Bias risk assessment for prevalent studies based on JBI.docx]

**Table S4** Bias risk assessment for prevalent studies based on JBI’s scales

| Study | Q1 | Q2 | Q3 | Q4 | Q5 | Q6 | Q7 | Q8 | Q9 | Total scores |
| --- | --- | --- | --- | --- | --- | --- | --- | --- | --- | --- |
| Chunling Yang-2022 | 1 | 1 | 1 | 1 | 1 | 1 | 0 | 1 | NA | 7 |
| He Ye-2019 | 1 | 1 | 1 | 1 | 0 | 1 | 1 | 1 | 1 | 8 |
| He Ye-2020 | 1 | 0 | 0 | 1 | NA | 1 | 1 | 1 | NA | 5 |
| Junyang Yang-2019 | 1 | 1 | 1 | 1 | 0 | 1 | 1 | 1 | 1 | 8 |
| Li Hong-2014 | 1 | NA | 1 | 1 | 0 | 1 | 0 | 1 | 1 | 6 |
| Li Xiaodi-2022 | 1 | NA | 1 | 1 | 0 | 1 | 1 | 1 | 0 | 5 |
| Liang Linhui-2018 | 1 | NA | 1 | 1 | NA | 1 | 1 | 1 | 0 | 6 |
| Lili Dai-2020 | 1 | NA | 1 | 1 | 0 | 1 | 1 | 1 | 1 | 7 |
| Liu Min-2021 | 1 | NA | 1 | 1 | 1 | 1 | 0 | 1 | 1 | 7 |
| Liu Yanan-2021 | 1 | NA | 1 | 1 | NA | 1 | 0 | 1 | NA | 5 |
| Liu Yanan-2021 | 1 | NA | 1 | 1 | NA | 1 | 0 | 1 | NA | 5 |
| Ma Zhong-hui-2021 | 1 | NA | 1 | 1 | 1 | 1 | 0 | 1 | 1 | 7 |
| Qi Wang-2016 | 1 | NA | 1 | 1 | 1 | 1 | 0 | 1 | 0 | 6 |
| Teng Hu-2020 | 1 | NA | 1 | 1 | 1 | 1 | 0 | 1 | 0 | 6 |
| Wang Yin-2018 | 1 | NA | 1 | 1 | 0 | 1 | 0 | 1 | 0 | 5 |
| Xiao Jing-2021 | 1 | NA | 1 | 1 | 1 | 1 | 0 | 1 | 0 | 6 |
| Yan Dongmei-2021 | 1 | NA | 1 | 1 | 0 | 1 | 0 | 1 | 0 | 5 |
| Zhai You-2021 | 1 | NA | 1 | 1 | NA | 1 | 0 | 1 | NA | 5 |
| Zhao Yanwei-2019 | 1 | NA | 1 | 1 | 0 | 1 | 0 | 1 | 1 | 6 |
| Qi mingxue-2024 | 1 | NA | 1 | 1 | 1 | 1 | NA | 1 | NA | 6 |
| Zhang xiongle-2023 | 1 | NA | 1 | 1 | 1 | 1 | NA | 1 | NA | 6 |
| Luo Yanhe-2023 | 1 | NA | 1 | 1 | 1 | 1 | 0 | 1 | 1 | 7 |
| Kong deyong-2023 | 1 | NA | 1 | 1 | 1 | 1 | NA | 1 | NA | 6 |
| Zheng Tian-2023 | 1 | NA | 1 | 1 | 1 | 1 | 1 | 1 | 1 | 8 |
| Jia Huangchao-2023 | 1 | NA | 1 | 1 | 1 | 1 | NA | 1 | 1 | 7 |
| Xiaolei Wang-2023 | 1 | NA | 1 | 1 | 1 | 1 | NA | 1 | 1 | 7 |
| Xiuxia Li-2023 | 1 | NA | 1 | 1 | 1 | 1 | 1 | 1 | 1 | 8 |
| Chen Zhaoyun-2024 | 1 | NA | 1 | 1 | 1 | 1 | NA | 1 | NA | 6 |
| Chen Zhaoyun-2025 | 1 | NA | 1 | 1 | 1 | 1 | 1 | 1 | NA | 7 |
| Kou Cuiping-2025 | 1 | NA | 1 | 1 | 1 | 1 | NA | 1 | NA | 6 |
| Li Zhihui-2024 | 1 | NA | 1 | 1 | 1 | 1 | NA | 1 | NA | 6 |
| Lin Jing-2024 | 1 | NA | 1 | 1 | 1 | 1 | NA | 1 | 1 | 7 |
| Liu Yihan-2024 | 1 | NA | 1 | 1 | 1 | 1 | 1 | 1 | NA | 7 |
| Lei Li-2024 | 1 | NA | 1 | 1 | 1 | 1 | NA | 1 | NA | 6 |
| Yanan Wang-2024 | 1 | NA | 1 | 1 | 1 | 1 | NA | 1 | NA | 6 |
| Yide Xu-2024 | 1 | NA | 1 | 1 | 1 | 1 | NA | 1 | NA | 6 |

Notes：0=No; 1=Yes; NA=uncertain.

Q1:Was the sample frame appropriate to address the target population? Q2:Were study participants sampled in an appropriate way? Q3:Was the sample size adequate? Q4:Were the study subjects and the setting described in detail? Q5:Was the data analysis conducted with sufficient coverage of the identified sample? Q6:Were valid methods used for the identification of the condition? Q7:Was the condition measured in a standard, reliable way for all participants? Q8:Was there appropriate statistical analysis? Q9:Was the response rate adequate, and if not, was the low response rate managed appropriately?
