# Supplementary material for: The prevalence of dyslipidemia and its correlation with anti-retroviral therapy among people living with HIV in China: a systematic review and meta-analysis
Source: Front Cardiovasc Med. 2025 Jun 13;12:1498165. doi: 10.3389/fcvm.2025.1498165 (PMC12202347; doi:10.3389/fcvm.2025.1498165)
Supplement: Supplementary file 1 [file Datasheet1.zip › Supplementary Tables.zip!/Table S7 Citation of included articles.docx]

**Table S7 Citation of included articles**

| **Study** | **Citation(Origin)** |
| --- | --- |
| He Ye-2020 | YE H, HONG Y M, JING S X, et al. Widespread use of anti-retroviral therapy elevated the prevalence of dyslipidemia in HIV-infected Chinese patients[J/OL]. Brazilian Journal of Pharmaceutical Sciences, 2020, 56. https://doi.org/10.1590/S2175-97902020000318850. |
| Lili Dai-2020 | DAI L, SU B, LIU A, et al. Adverse events in Chinese human immunodeficiency virus (HIV) patients receiving first line antiretroviral therapy[J/OL]. BMC Infectious Diseases, 2020, 20(1). https://doi.org/10.1186/s12879-020-4878-2. |
| Xiao Jing-2021 | 肖静, 韩俊燕, 栗翠林,等. 抗逆转录病毒治疗时长对HIV/AIDS患者机体代谢的影响[J]. 中华实验和临床病毒学杂志, 2021, 35(2). |
| Wang Yin-2018 | 王印, 王亚丽, 叶江竹, 等. 以EFV为基础的初始抗病毒治疗方案对成人HIV/AIDS病人血脂的影响[J/OL]. 中国艾滋病性病, 2018, 24(08): 768-771. https://doi.org/10.13419/j.cnki.aids.2018.08.05. |
| Qi Wang-2016 | WANG Q, DING H, XU J, et al. Lipids profile among ART-naïve HIV infected patients and men who have sex with men in China: a case control study[J/OL]. Lipids in Health and Disease, 2016, 15(1): 149. https://doi.org/10.1186/s12944-016-0297-1. |
| He Ye-2019 | 何叶, 余旻虹, 宋晓璟, 等. 高效抗逆转录病毒治疗HIV/AIDS患者血脂水平与饮食习惯及血脂知识的研究[J]. 护理学杂志, 2019, 34(18). |
| Zhao Yanwei-2019 | 赵艳伟, 宋晓璟, 余旻虹, 等. HIV/AIDS患者体力活动现状及血脂异常影响因素研究[J]. 中国护理管理, 2019, 19(12): 1770-1774. |
| Liu Min-2021 | 刘敏, 石峰, 刘倩, 等. 依非韦伦和洛匹那韦/利托那韦对成人艾滋病患者血脂水平影响的对比研究[J/OL]. 中华传染病杂志, 2021, 39(3). |
| Liang Lin-hui-2018 | 梁林慧, 余飞, 黄承乐, 等. 人类免疫缺陷病毒感染者高效抗逆转录病毒治疗后生化计量指标的变迁[J]. 当代医学, 2018, 24(11): 5-8. |
| Zhai You-2021 | 翟优, 赵英强, 刘亚楠, 等. 河南某地区经性传播途径感染艾滋病患者高脂血症患病率及影响因素分析[J/OL]. 中国皮肤性病学杂志, 2021, 35(8): 897-903. https://doi.org/10.13735/j.cjdv.1001-7089.202102013. |
| Liu Ya-nan-2021a | 刘亚楠, 马彦民, 陈莉华, 等. 河南某地女性HIV/AIDS患者高脂血症及影响因素分析[J/OL]. 中国皮肤性病学杂志, 2021, 35(6): 646-650+669. https://doi.org/10.13735/j.cjdv.1001-7089.202012101 |
| Liu Ya-nan-2021b | 刘亚楠, 马彦民, 刘志斌, 等. 真实世界1537例性传播HIV/AIDS患者高脂血症患病率及影响因素[J]. 中华中医药杂志, 2021, 36(5): 2683-2687. |
| Chunling Yang-2022 | YANG C, WANG D, MA Y, 等. Effectors of Hyperlipidemia Among Patients with HIV/AIDS Taking Second-line Antiretroviral Therapy Based on Registry Data[J/OL]. Current HIV Research, 2022, 20(5): 373-379.  https://doi.org/10.2174/1570162X20666220805103411. |
| Li Hong-2014 | 李虹, 陈媛媛, 何云, 等. 两种ART方案对HIV/AIDS患者血脂的影响[J]. 重庆医学, 2014, 43(20): 2574-2576. |
| Li Xiao-di-2022 | 李小迪, 曹玮, 刘正印, 等. 长期联合抗反转录病毒治疗对人类免疫缺陷病毒感染/艾滋病患者心血管疾病风险的影响[J/OL]. 中华传染病杂志, 2022, 40(8). |
| Junyang Yang-2019 | YANG J, CHEN J, JI Y, et al. Lipid profile and renal safety of tenofovir disoproxil fumarate-based anti-retroviral therapy in HIV-infected Chinese patients[J/OL]. International Journal of Infectious Diseases, 2019, 83: 64-71. https://doi.org/10.1016/j.ijid.2019.03.034. |
| Yan Dongmei-2021 | 严冬梅,王宗正,蔡琳,等. TLE方案对初治男男性行为者的HIV/AIDS患者血脂及致动脉粥样硬化率的影响[J/OL]. 中国艾滋病性病, 2021, 27(1): 21-24. https://doi.org/10.13419/j.cnki.aids.2021.01.06. |
| Teng Hu-2020 | HU T, SI G, RONG X, et al. Correlation between cardiovascular risk factors in HIV-infected patients and three highly active anti-retroviral therapy regimens[J]. Int J Clin Exp Med 2020;13(2):988-994. |
| Ma Zhonghui-2021 | MA Z H, GAO J, CAO D D, 等. Prevalence and associated factors of multimorbidity among newly reported HIV-positive individuals in Dehong Dai and Jingpo Autonomous Prefecture[J/OL]. Chinese Journal of Disease Control and Prevention, 2021, 25(12): 1380-1386.  https://doi.org/10.16462/j.cnki.zhjbkz.2021.12.004 |
| Qi Mingxue-2024 | 祁明雪,苏一帆,陈晨,等. 依非韦伦、奈韦拉平或洛匹那韦/利托那韦方案对艾滋病病毒/艾滋病病人血脂代谢的长期影响[J]. 安徽医药,2024,28(5):1017-1024. https://doi.org/10.3969/j.issn.1009-6469.2024.05.036. |
| Zhang Xiongle-2023 | 张雄乐,陈小清,陈芬兰.福清地区3TC+TDF+EFV方案对HIV患者血脂水平影响的研究[J].海峡药学, 2023, 35(8):96-99. |
| Luo Yanhe-2023 | 罗燕和,孟小聖,余晓丽,等.武汉市HIV/AIDS患者抗病毒治疗前后血脂变化及影响因素[J].公共卫生与预防医学, 2023, 34(5):112-115. |
| Kong Deyong-2023 | 孔德勇,缪星国,苏菲菲,等.青年HIV/AIDS患者血脂异常的相关危险因素分析[J].中国皮肤性病学杂志,2023,37(8):941-946.  https://doi.org/10.13735/j.cjdv.1001-7089.202303010. |
| Zheng Tian-2023 | 郑甜,邓江玲,李树涛,等.比较不同抗逆转录病毒治疗方案对老年HIV感染者血脂水平的影响[J].中国性科学,2023,32(11):152-156.  https://doi.org/10.3969/j.issn.1672-1993.2023.11.038. |
| Jia huangchao-2023 | 贾皇超,郭会军,王东利,等.河南省艾滋病患者转服二线抗病毒治疗方案后血脂异常的发生率及影响因素分析[J].中国中西医结合杂志,2023,43(7):817-821. https://doi.org/10.7661/j.cjim.20230117.066. |
| Xiaolei Wang-2023 | Wang, X., Xiao, J., Zhang, L., et al. (2023). Longitudinal analysis of immune reconstitution and metabolic changes in women living with HIV: A real-world observational study. Chinese medical journal, 136(18), 2168–2177. https://doi.org/10.1097/CM9.0000000000002756 |
| Xiuxia Li-2023 | Li, X., Song, X., Han, Y., Qiu, Z., Cao, W., & Li, T. (2023). Risk factors and longitudinal changes of dyslipidemia among Chinese people living with HIV receiving antiretroviral therapy. BMC infectious diseases, 23(1), 598. https://doi.org/10.1186/s12879-023-08587-0 |
| Chen Zhaoyun-2024 | 陈昭云,杨萱,赵淑娴,等.不同抗病毒治疗方案对HIV/AIDS患者血脂的影响[J].中国艾滋病性病,2024,30(12):1247-1252.  DOI:10.13419/j.cnki.aids.2024.12.03. |
| Chen Zhaoyun-2025 | 陈昭云,赵清霞,杨萱,等. 河南省2023年HIV感染者常见慢性病患病情况及相关因素分析[J]. 中华流行病学杂志,2025,46(02)：258-263.  DOI:10.3760/cma.j.cn112338-20240626-00375 |
| Kou Cuiping-2025 | 寇翠萍,何琼,陈发青,等.HIV/AIDS高脂血症患者抗逆转录病毒治疗6年后血脂血糖监测结果分析[J].预防医学情报杂志,2024,40(09):1163-1167.DOI:10.19971/j.cnki.1006-4028.240291. |
| Li Zhihui-2024 | 李智慧,沙敏,杨磊,等.HIV/AIDS患者血脂异常、高血糖患病率及影响因素分析[J].中国性科学,2024,33(12):135-139. |
| Lin Jing-2024 | 林婧,邱园园,李粤平,等.HIV/AIDS患者ART过程中高三酰甘油血症、高胆固醇血症、高血糖发生情况及其影响因素[J].广东药科大学学报,2024,40(04):130-138.DOI:10.16809/j.cnki.2096-3653.2024030603. |
| Liu Yihan-2024 | 刘怡涵,刘俊,金永梅,等.HIV抗病毒治疗患者心血管疾病风险评估及危险因素分析[J].昆明医科大学学报,2024,45(10):140-146. |
| Lei Li-2024 | Li L, Zhang Y, Yu A, et al. Prevalence of dyslipidemia and associated risk factors among Chinese people living with HIV. *Chin Med J (Engl)*. 2024;137(23):2874-2876. doi:10.1097/CM9.0000000000003336 |
| Yanan Wang-2024 | Wang Y, Yang Z, Li J, et al. The risk of dyslipidemia on PLHIV associated with different antiretroviral regimens in Huzhou. *PLoS One*. 2024;19(9):e0305461. Published 2024 Sep 20.  doi:10.1371/journal.pone.0305461 |
| Yide Xu-2024 | Xu Y, Qi B, Wu X, et al. Interaction between antiretroviral therapy regimens and body mass index on triglyceride levels in people living with HIV: a cross-sectional and longitudinal study. *Lipids Health Dis*. 2024;23(1):232. Published 2024 Jul 30. doi:10.1186/s12944-024-02224-8 |
| Sun Liqin-2022 | Sun L, He Y, Xu L, et al. Higher Risk of Dyslipidemia With Coformulated Elvitegravir, Cobicistat, Emtricitabine, and Tenofovir Alafenamide than Efavirenz, Lamivudine, and Tenofovir Disoproxil Fumarate Among Antiretroviral-Naive People Living With HIV in China. J Acquir Immune Defic Syndr. 2022 Oct 1;91(S1):S8-S15.  https://doi.org/10.1097/QAI.0000000000003040. |
| Xiao Jiang-2011 | 肖江, 郜桂菊, 李鑫, 等. 高效抗逆转录病毒治疗对艾滋病患者机体代谢的影响. 中华实验和临床感染病杂志(电子版) 2011, 5, 133–141. |
| Zhang Yong-2011 | 张勇, 卢瑞朝, 苏凌松, 等.含克力芝组合的HAART方案对HIV/HCV混合感染的疗效探讨. 中国艾滋病性病,2011, 17, 633–635, https://doi.org/10.13419/j.cnki.aids.2011.06.019. |
| Yang Zhibin-2012 | 杨智彬, 雷玉萍, 曾荣昆, 等.高效抗反转录病毒疗法对艾滋病患者血脂的影响. 临床内科杂志,2012, 29. |
| Zhao xiangmei-2012 | 赵香梅, 孙挥宇, 陈凤欣, 等. 获得性免疫缺陷综合征患者血脂和眼底动脉硬化的研究. 临床荟萃, 2012, 27, 1214–1217. |
| Li Yanqing-2020 | 李燕青, 简凤璧, 谭俊.艾滋病患者HAART治疗前后CD4^+T淋巴细胞及血液生化指标变化分析. 今日药学，2020, 30. |
| Teng caiwan-2019 | 滕采万, 何茂锐, 赵渝琼, 等. 高效抗逆转录病毒治疗对AIDS患者机体代谢及T淋巴细胞亚群的影响. 贵州医科大学学报 2019, 44, 712-715+720. https://doi.org/10.19367/j.cnki.1000-2707.2019.06.019. |
| Li Xiaodi-2022 | 李小迪, 曹玮, 刘正印, 等. 长期联合抗反转录病毒治疗对人类免疫缺陷病毒感染/艾滋病患者心血管疾病风险的影响 [J] . 中华传染病杂志, 2022, 40(8) : 496-504. DOI: 10.3760/cma.j.cn311365-20211118-00404. |
| Liang Linhui-2018 | 梁林慧,余飞,黄承乐,等.人类免疫缺陷病毒感染者高效抗逆转录病毒治疗后生化计量指标的变迁[J].当代医学,2018,24(11):5-8. |
| Yan Dongmei-2022 | 严冬梅,杨彤彤,何沅鸿,等.不同剂量依非韦伦对成都地区初治AIDS患者血脂及血浆致动脉硬化指数的影响[J].重庆医学,2022,51(22):3832-3835. |
| Xi Wang-2024 | Wang X, Liu A, Li Z, et al. Changes in blood lipid levels and influencing factors among treatment-naïve adult male HIV/AIDS patients following BIC/FTC/TAF vs . 3TC+EFV+TDF. *Chin Med J (Engl)*. 2024;137(12):1447-1452. doi:10.1097/CM9.0000000000003147 |
