# Supplementary material for: The prevalence of dyslipidemia and its correlation with anti-retroviral therapy among people living with HIV in China: a systematic review and meta-analysis
Source: Front Cardiovasc Med. 2025 Jun 13;12:1498165. doi: 10.3389/fcvm.2025.1498165 (PMC12202347; doi:10.3389/fcvm.2025.1498165)
Supplement: Supplementary file 1 [file Datasheet1.zip › Supplementary Tables.zip!/Table S8 Characteristics of included studies.docx]

**Table S8** Characteristics of included studies

| Study (first author and year of publication) | content | Design | Region | Time of recruitment | Sample size | Female (%) | Mean(±SD)/Median(IQR)/Range of age | Mean/Median of ART duration（months） | ART regimens | Diagnostic guideline | Criteria |
| --- | --- | --- | --- | --- | --- | --- | --- | --- | --- | --- | --- |
|  |  |  |  |  |  |  |  |  |  |  |  |
|  |  |  |  |  |  |  |  |  |  |  |  |
| Chunling Yang-2022 | Prevalence | Cross-sectional | Henan | 2018 | 2886 | 55.1 | 51.4±7.7 | — | — | Guidelines on Prevention and Treatment of Blood Lipid Abnormality in Chinese Adults(2016) | TC>6.2mmol/L;TG>2.26mmol/L;LDL>4.14mmol/L;HDL<1.04mmol/L |
| He Ye-2019 | Prevalence | Cross-sectional | Beijing | 2017-2018 | 306 | 9.8 | 36±7.5 | — | — | Guidelines on Prevention and Treatment of Blood Lipid Abnormality in Chinese Adults(2016 Adjusted) | TC>5.2mmol/L;TG>1.7mmol/L;LDL>3.4mmol/L;HDL<1.0mmol/L |
| He Ye-2020 | Prevalence | Cross-sectional | Beijing | 2016 | 128 | 10.2 | 38.8±11 | — | — | 2001 NCEP-ATP Ⅲ | TC>5.70mmol/L;TG>1.70mmol/L;LDL-C>2.50mmol/L;HDL-C<1.0mmol/L |
| Junyang Yang-2019 | Prevalence | Cohort | Shanghai | 2013-2015 | 414 | 3.4 | 35.33 ±11.09 | 24 | NRTIs+NNRTI | 2001 NCEP-ATP Ⅲ | TC>5.70mmol/L;TG>1.70mmol/L;LDL-C>2.50mmol/L;HDL-C<1.0mmol/L |
| Li Hong-2014 | Prevalence | Cohort | Henan | 2009-2011 | 102 | 42.2 | 18-65 | 12 | NNRTIs+NRTI | Guidelines on Prevention and Treatment of Blood Lipid Abnormality in Chinese Adults(2007) | TC>5.18mmol/L;TG>1.70mmol/L;LDL>3.37mmol/L;HDL<1.04mmol/L |
| Li Xiaodi-2022 | Both | Case-control | Multiple centers | 2018-2020 | 601 | 18.5 | 39.8 | >60 | — | 2001 NCEP-ATP Ⅲ | TC>5.18mmol/L;TG>1.70mmol/L;LDL>3.37mmol/L;HDL<1.04mmol/L |
| Liang Linhui-2018 | Prevalence | Cohort | Guangxi | 2013-2016 | 300 | — | — | 36 | — | Guidelines on Prevention and Treatment of Blood Lipid Abnormality in Chinese Adults(2016) | TC>6.2mmol/L;TG>2.26mmol/L;LDL>4.14mmol/L;HDL<1.04mmol/L |
| Lili Dai-2020 | Prevalence | Cohort | Beijing | 2017 | 474 | 2.3 | 34.0 ± 10.1 | 12 | NNRTI+NRTIs | DAIDS 2017 | TC>5.18mmol/L;TG>1.71mmol/L;LDL>3.37mmol/L |
| Liu Min-2021 | Both | Cohort | Chongqing | 2015-2018 | 272 | 21.0 | 39.2±13.2 | 12 | NRTIs+NNRTI/LPV/r | 2001 NCEP-ATP Ⅲ | TC>6.20 mmol /L;TG >2.30 mmol/L |
| Liu Yanan-2021a | Prevalence | Cross-sectional | Henan | 2018 | 1537 | 39.9 | 47.7±9.9 | — | — | Guidelines on Prevention and Treatment of Blood Lipid Abnormality in Chinese Adults(2016 Adjusted) | TC>6.20 mmol /L;TG >2.30 mmol/L |
| Liu Yanan-2021b | Prevalence | Cross-sectional | Henan | 2018 | 1538 | 100.0 | 49.36±8.84 | — | — | Guidelines on Prevention and Treatment of Blood Lipid Abnormality in Chinese Adults(2016 Adjusted) | TC>6.20 mmol /L;TG >2.30 mmol/L |
| Ma Zhonghui-2021 | Prevalence | Cross-sectional | Yunnan | 2018-2020 | 468 | 42.1 | — | — | — | Guideline for primary care of dyslipidemias (2019) | TC>6.20 mmol /L;TG >2.30 mmol/L |
| Qi Wang-2016 | Prevalence | Cross-sectional | Beijing | 2013-2015 | 220 | 0.0 | — | — | — | WHO | TC>6.20 mmol /L;TG >2.30 mmol/L |
| Teng Hu-2020 | Both | Cohort | Sichuan | 2016-2017 | 108 | 39.8 | 46.6 | 12 | NRTIs+NNRTI/LPV/r | — | TC>6.20 mmol /L;TG >2.30 mmol/L |
| Wang Yin-2018 | Prevalence | Cohort | Beijing | 2013-2015 | 110 | 0.0 | 32(28,38) | 12 | NRTIs+NNRTI | Guidelines on Prevention and Treatment of Blood Lipid Abnormality in Chinese Adults(2016) | TC>6.20 mmol /L;TG >2.30 mmol/L |
| Xiao Jing-2021 | Prevalence | Cross-sectional | Beijing | 2020 | 424 | 1.9 | 34(30,40) | 41 | — | 2001 NCEP-ATP Ⅲ | TC>6.20 mmol /L;TG >2.30 mmol/L |
| Yan Dongmei-2021 | Both | Cohort | Sichuan | 2014-2017 | 209 | 0.0 | 18-47 | 24 | — | Guidelines on Prevention and Treatment of Blood Lipid Abnormality in Chinese Adults(2016 Adjusted) | TC ≥ 5.2 mmol/L; TG≥ 1.7 mmol/L ; LDL-C≥ 3.37 mmol/L; HDL-C< 1.04 mmol/L |
| Zhai You-2021 | Prevalence | Cross-sectional | Henan | 2018 | 1020 | 39.6 | — | — | — | Guidelines on Prevention and Treatment of Blood Lipid Abnormality in Chinese Adults(2016 Adjusted) | TC ≥ 5.2 mmol/L; TG≥ 1.7 mmol/L ; LDL-C≥ 3.37 mmol/L; HDL-C< 1.04 mmol/L |
| Zhao Yanwei-2019 | Prevalence | Cross-sectional | Beijing | 2017-2019 | 306 | 9.8 | 38.83±11.05 | — | — | Guidelines on Prevention and Treatment of Blood Lipid Abnormality in Chinese Adults(2016 Adjusted) | TC ≥ 5.2 mmol/L; TG≥ 1.7 mmol/L ; LDL-C≥ 3.37 mmol/L; HDL-C< 1.04 mmol/L |
| Qi mingxue-2024 | Prevalence | Cohort | jiangsu | 2013-2021 | 1321 | 4.7 | 31(25,40) | 24 | NRTIs+NNRTI/LPV/r | 2001 NCEP-ATP Ⅲ | TC>6.20 mmol /L;TG >2.30 mmol/L |
| Zhang xiongle-2023 | Prevalence | Cohort | Fujian | -2022.12 | 184 | 20.1 | 44(33,56) | 38 | — | - | TC ≥ 5.2 mmol/L; TG≥ 1.7 mmol/L ; LDL-C≥ 3.37 mmol/L; HDL-C< 1.04 mmol/L |
| Luo Yanhe-2023 | Prevalence | Cohort | Hubei | 2004-2021 | 7546 | 9.4 | 38.1±14.97 | — | — | - | TC>6.20 mmol /L;TG >2.30 mmol/L |
| Kong deyong-2023 | Prevalence | Cohort | Zhejiang | 2022.2-7 | 217 | 8.8 | 18-44 | — | NRTIs+NNRTI/LPV/r/INSTI | Guidelines on Prevention and Treatment of Blood Lipid Abnormality in Chinese Adults(2016 Adjusted) | TC≥5.2 mmol / L; TG≥ 1.7 mmol/L; LDL-C≥3. 4 mmol/L; HDL-C< 1. 0 mmol/L |
| Zheng Tian-2023 | Both | RCT | Xinjiang | 2019-2021 | 104 | 9.6 | 71.62±5.63 | 12 | NRTIs+NNRTI LPV/r | - | TC ≥ 5.2 mmol/L; TG≥ 1.7 mmol/L; LDL-C≥ 3.37 mmol/L; HDL-C< 1.04 mmol/L |
| Jia Huangchao-2023 | Prevalence | Cohort | Henan | 2009- 2015 | 2086 | 49.6 | 0 | — | — | Guidelines on Prevention and Treatment of Blood Lipid Abnormality in Chinese Adults(2016 Adjusted) | TC>6.20 mmol /L;TG >2.30 mmol/L |
| Xiaolei Wang-2023 | Prevalence | Cohort | Beijing | 2005-2021 | 301 | 100.0 | 37 (28,48) | 49.2 | — | 2001 NCEP-ATP Ⅲv | TC ≥5.20 mmol/L; TG ≥1.70 mmol/L; LDL-C ≥3.37 mmol/L; HDL-C <1.04 mmol/L, |
| Xiuxia Li-2023 | Prevalence | Cohort | Multiple centers | — | 1542 | 19.6 | 35.8 ± 10.4 | 72 | — | 2001 NCEP-ATP Ⅲ | TC ≥ 5.2 mmol/L; TG≥ 1.7 mmol/L; LDL-C≥ 3.37 mmol/L; HDL-C< 1.04 mmol/L |
| Chen Zhaoyun-2024 | Prevalence | Cohort | Henan | 2023.1-12 | 2312 | 14.1 | 43.34±13.60 | ≥6 | NRTIs+EFV/LPV/r/INSTI | Chinese guidelines for lipid management (2023) | TC≥5.2 mmol/L、TG≥1.7 mmol/L、 HDL-C<1.0 mmol/L、LDL-C≥3.4 mmol/L |
| Chen Zhaoyun-2025 | Prevalence | Cohort | Henan | -2023.12 | 4023 | 14.7 | 43.7±14.0 | 0 | — | Chinese guidelines for lipid management (2023) | TC>6.2mmol/L;TG>2.26mmol/L;LDL>4.14mmol/L;HDL<1.04mmol/L |
| Kou Cuiping-2025 | Prevalence | Cross-sectional | Gansu | 2017 | 400 | 22.8 | 46.34 ± 10.34 | 84 | — | 0 | TC≥6.20 mmol/L，TG≥2.30 mmol/L |
| Li Zhihui-2024 | Prevalence | Cross-sectional | Yunnan | 2011-2021 | 156 | 47.4 | (38.6±9.6) | 120 | — | 2001 NCEP-ATP Ⅲv | TC≥5.2mmol/L，TG≥1.7mmol/L |
| Lin Jing-2024 | Prevalence | Cohort | Guangdong | 2004-2019 | 8839 | 16.3 | — | 0 | — | Guidelines on Prevention and Treatment of Blood Lipid Abnormality in Chinese Adults(2016 Adjusted) | TC ≥ 6.2 mmol/L，TG ≥ 2.3 mmol/L |
| Liu Yihan-2024 | Prevalence | Cohort | Yunnan | 2023.7-12 | 4139 | 38.2 | — | 0 | — | 0 | TC>6.2mmol/L;TG>2.26mmol/L;LDL>4.14mmol/L;HDL<1.04mmol/L |
| Lei Li-2024 | Prevalence | Cohort | Multiple centers | 0 | 5211 | 20.8 | 43.2 ± 13.7 | 0 | — | 2001 NCEP-ATP Ⅲv | TC ≥5.20 mmol/L, HDL-C <1.04 mmol/L, LDL-C ≥3.37 mmol/L, or TG ≥1.70 mmol/L. |
| Yanan Wang-2024 | Prevalence | Cohort | Zhejiang | 2005.7-2022.6 | 532 | 13.9 | 18–82 | 0 | NRTIs+NNRTI | Guidelines on Prevention and Treatment of Blood Lipid Abnormality in Chinese Adults(2016 Adjusted) | TC≥ 5.2mmol/L or TG ≥1.7mmol/L or LDL-C≥ 3.37mmol/L or HDL-C≥ 1.04mmol/L. |
| Yide Xu-2024 | Prevalence | Cross-sectional & Cohort | Jiangsu | 2021-2023 | 451 | 0.0 | 36.0 (32.0–44.0) | 0 | NRTIs+NNRTI | — | TG≥1.7mmol/L. |
| Sun Liqin-2022 | MD | Cohort | Guangdong | 2018-2020 | 344 | 9.0 | — | 12 | NRTIs+NNRTI/INSTI | — | — |
| Xiao Jiang-2011 | MD | Cohort | Guangdong | 2014-2018 | 83 | — | — | 0 | — | — | — |
| Zhang Yong-2011 | MD | Cohort | Guangxi | 2008.10-2009.10 | 43 | 16.3 | 35.5±6.1 | 12 | NRTIs+LPV/r | — | — |
| Yang Zhibin-2012 | MD | Cohort | Yunnan | 2005-2011 | 100 | 43.0 | — | 40.78 | NRTIs+NNRTI | — | — |
| Zhao xiangmei-2012 | MD | Cohort | Beijing | 2009.6-2011.11 | 100 | 21.0 |  |  |  | — | — |
| Li Yanqing-2020 | MD | Cohort | Guangdong | 2018-2018 | 131 | 32.1 | — | 21 | — | — | — |
| Teng caiwan-2019 | MD | RCT | Chongqing | 2015-2018 | 48 | 65.2 | 38.6±3.3 | 12 | NRTIs+NNRTI/LPV/r | — | — |
| Yan Dongmei-2022 | MD | RCT | SIchuan | 2016.5-2018.3 | 46 | 0.0 | 19-43 | 12 | NRTIs+EFV | — | — |
| Xi Wang-2024 | MD | Cohort | Beijing | 2021.1-12 | 870 | — | — | 12 | NRTIs+NRTIs/INSTI | — | — |

Note: 2001 NCEP-ATP Ⅲ = National Cholesterol Education Program (NCEP) Adult Treatment Panel (ATP) III
