# Supplementary material for: The prevalence of dyslipidemia and its correlation with anti-retroviral therapy among people living with HIV in China: a systematic review and meta-analysis
Source: Front Cardiovasc Med. 2025 Jun 13;12:1498165. doi: 10.3389/fcvm.2025.1498165 (PMC12202347; doi:10.3389/fcvm.2025.1498165)
Supplement: Supplementary file 1 [file Datasheet1.zip › Supplementary Tables.zip!/Table S5 Bias risk assessment for cohorts reporting mean difference of serum lipids.docx]

**Table S5** Bias risk assessment for cohorts reporting mean difference of serum lipids

| title | A1 | A2 | A3 | A4 | B | C1 | C2 | C3 | Total |
| --- | --- | --- | --- | --- | --- | --- | --- | --- | --- |
| Sun Liqin-2022 | 1 | 1 | 1 | 0 | 1 | 1 | 1 | 1 | 8 |
| Xiao Jiang-2011 | 1 | 1 | 1 | 1 | 0 | 1 | 1 | 1 | 8 |
| Zhang Yong-2011 | 1 | 1 | 1 | 1 | 1 | 1 | 1 | 1 | 9 |
| Yang Zhibin-2012 | 0 | 1 | 1 | 0 | 0 | 1 | 1 | 1 | 7 |
| Zhao xiangmei-2012 | 1 | 1 | 1 | 0 | 1 | 1 | 1 | 1 | 8 |
| Li Yanqing-2020 | 1 | 1 | 1 | 0 | 1 | 1 | 1 | 1 | 8 |
| Liu MIn-2021 | 1 | 1 | 1 | 0 | 0 | 1 | 1 | 1 | 7 |
| Li Xiaodi-2022 | 1 | 1 | 1 | 0 | 1 | 1 | 1 | 1 | 8 |
| Teng Hu-2020 | 1 | 1 | 1 | 1 | 1 | 1 | 1 | 1 | 9 |
| Xi Wang-2024 | 1 | 1 | 1 | 0 | 1 | 1 | 1 | 1 | 8 |

Notes：0=No; 1=Yes; NA=uncertain.

A1：Representativeness of the exposed cohort

A2: Selection of the non exposed cohort

A3: Ascertainment of exposure

A4: Demonstration that outcome of interest was not present at start of study

B: Comparability of cohorts on the basis of the design or analysis

C1: Assessment of outcome

C2: Was follow-up long enough for outcomes to occur

C3: Adequacy of follow up of cohorts.
