# Supplementary material for: The prevalence of dyslipidemia and its correlation with anti-retroviral therapy among people living with HIV in China: a systematic review and meta-analysis
Source: Front Cardiovasc Med. 2025 Jun 13;12:1498165. doi: 10.3389/fcvm.2025.1498165 (PMC12202347; doi:10.3389/fcvm.2025.1498165)
Supplement: Supplementary file 1 [file Datasheet1.zip › Supplementary Tables.zip!/Table S6 Bias risk assessment for RCTs reporting mean difference of serum lipids.docx]

**Table S6** Bias risk assessment for RCTs reporting mean difference of serum lipids

| Study | Q1 | Q2 | Q3 | Q4 | Q5 | Q6 | Q7 | Q8 | Q9 | Q10 | Q11 | Q12 | Q13 | Total |
| --- | --- | --- | --- | --- | --- | --- | --- | --- | --- | --- | --- | --- | --- | --- |
| Teng caiwan-2019 | 1 | NA | NA | 1 | NA | NA | 1 | 1 | 1 | 1 | 1 | 1 | 0 | 8 |
| Yan Dongmei-2022 | 1 | NA | 1 | 1 | NA | NA | 1 | 1 | 1 | 1 | 1 | 1 | 0 | 9 |
| Zheng Tian-2023 | 1 | NA | 1 | 1 | NA | NA | 1 | 1 | 1 | 1 | 1 | 1 | 0 | 9 |

Note: 0=No; 1=Yes; NA=uncertain. Q1. Was true randomization used for assignment of participants to treatment groups?

Q2. Was allocation to treatment groups concealed?

Q3. Were treatment groups similar at the baseline?

Q4. Were participants blind to treatment assignment?

Q5. Were those delivering treatment blind to treatment assignment?

Q6. Were outcomes assessors blind to treatment assignment?

Q7. Were treatment groups treated identically other than the intervention of interest?

Q8. Was follow up complete and if not, were differences between groups in terms of their follow up adequately described and analyzed?

Q9. Were participants analyzed in the groups to which they were randomized?

Q10. Were outcomes measured in the same way for treatment groups?

Q11. Were outcomes measured in a reliable way?

Q12. Was appropriate statistical analysis used?

Q13. Was the trial design appropriate, and any deviations from the standard RCT design (individual randomization, parallel groups) accounted for in the conduct and analysis of the trial?
